# Structural Damage Assessment and Resilience Evolution Prediction of Immersed Tunnels During Sand Foundation Loss Using In Situ Sensing Data

**DOI:** 10.3390/s26175358

**Published:** 2026-08-25

**Authors:** Weili Chen, Zequan Yu, Zhen Feng, Yadong Li, Baoping Chen

**Affiliations:** 1 Guangzhou Road Affairs Center, Guangzhou 510635, China; 2 Guangdong Jianke Traffic Engineering Quality Inspection Center Co., Ltd., Guangzhou 510500, China; 3 Guangdong Provincial Intelligent Testing Engineering Technology Research Center for Transportation Infrastructure, Guangzhou 510500, China; 4 School of Civil Engineering, Guangzhou University, Guangzhou 510006, China; 5 School of Architecture, Kashi University, Kashi 844000, China

**Keywords:** immersed tunnels, sand foundation loss, damage assessment, resilience evolution, in situ sensing, numerical simulations

## Abstract

**Highlights:**

**What are the main findings?**

- A joint sensing system combining DOFS for structural deformation monitoring and impact imaging for sand foundation defect detection is established to identify both structural damage and foundation deterioration.
- A redundancy-based resilience evaluation framework is established to provide quantitative indicators for foundation remediation.

**What are the implications of the main findings?**

- The integrated sensing scheme realizes synchronous identification of lining damage and foundation hidden defects under complex tunnel conditions.
- The quantitative resilience indicators can support decision-making for foundation reinforcement and remediation of immersed tunnels.

**Abstract:**

The loss of sand foundation often induces differential settlement in immersed tunnel segments, potentially causing structural damage and reducing structural resilience. Accurately assessing the damage characteristics and their effects on resilience during sand foundation loss is essential for ensuring tunnel safety. This study adopts a typical immersed tunnel project as a case study. Long-term structural deformation data acquired by distributed optical fiber sensing technology and sand foundation detection data are adopted to analyze the response characteristics and damage state of the tunnel. A refined three-dimensional tunnel–stratum interaction model is established and validated against monitoring data to investigate mechanical response characteristics, including deformation and bending moment distributions. A redundancy factor is proposed as a quantitative index for tunnel resilience under foundation loss, and a multi-level resilience grading framework is established accordingly. Furthermore, the evolution of tunnel resilience under various displacement recovery ratios, which represent the extent of differential settlement remediation, is investigated using the refined numerical model. Field detection results show that over 50% of the foundation area is affected by loosening or voids. These defects are highly consistent with regions of abnormal structural deformation, leading to a bending–torsional deformation mode, with a maximum joint differential settlement of 106.7 mm. Stress concentration occurs in the tunnel floor above denser sand zones, with a maximum crack width of 0.43 mm. The tunnel is classified as severely damaged (low resilience) based on the proposed standard, with a redundancy factor of 1.59. Bending-torsional deformation and stress concentration are gradually mitigated as the displacement recovery ratio increases. The redundancy factor exhibits a parabolic relationship with the recovery ratio, indicating that tunnel resilience can be restored to a relatively high level when the displacement recovery ratio exceeds 70%. The proposed redundancy factor and grading framework provide quantitative guidance for designing and optimizing resilience improvement strategies following sand foundation loss.

## 1. Introduction

Immersed tunnels have several advantages, including low subsoil bearing capacity requirements, efficient space utilization, and strong adaptability to complex hydrometeorological conditions [1]. As one of the critical underwater transportation technologies, immersed tunnels are widely used around the world. According to incomplete statistics, nearly 200 immersed tunnels have been constructed globally. Although immersed tunnels provide an effective means of crossing a waterway, various typical structural defects gradually appear due to the long-term coupling effects of self-weight, traffic loads, and hydraulic erosion [2,3,4,5,6]. The most prominent of these are segment cracking, joint shear key failure, and water leakage induced by differential settlement. These problems significantly undermine the resilience of immersed tunnels and pose a major threat to their operational safety [7].

The pre-bedded method and sand-flow method are two of the most widely used foundation construction methods for immersed tunnels. When using the pre-bedded method, foundation treatment is generally completed before the tunnel segments are floated and installed. However, with the sand-flow method, the tunnel segments are filled with sand only after the segments have been sunk and connected. Therefore, the compaction state of the sand foundation is a key factor governing the long-term service performance of immersed tunnels treated with the sand-flow method [8,9,10]. The loss of the sand foundation in tunnels constructed using the sand-flow method is one of the most significant and detrimental causes of differential settlement. This can result in loose zones forming in the foundation, and in severe cases, voids may form beneath the tunnel segments, which in turn triggers a redistribution of internal structural forces and stress concentration, thereby accelerating crack propagation and joint damage [10,11,12].

However, monitoring sand foundation conditions and structural deformation in immersed tunnels presents substantial technical challenges. The sand foundation is concealed beneath the tunnel in a complex underwater environment and is vulnerable to water erosion and sediment deposition. Traditional manual inspection and single-point monitoring approaches are limited to surface observation, with poor spatial coverage and a high risk of missing local foundation defects. With the rapid development of structural health monitoring (SHM) technology, various sensing techniques have been widely adopted throughout the construction and operation phases of immersed tunnels [13]. Among them, distributed optical fiber sensing is excellent for long-distance, continuous monitoring with high spatial resolution when measuring structural deformation. Meanwhile, impact imaging technology, which is based on elastic wave analysis, is highly precise and has strong penetration capabilities for detecting defects in underwater foundations. Combining the two sensing methods enables full-coverage monitoring of both structural responses and foundation conditions. Long-term monitoring data acquired using these techniques can effectively reflect the actual performance of tunnels in complex operating environments [14].

Existing studies on immersed tunnel sand foundations mainly concentrate on construction-related issues, including foundation treatment methods, sand flow characteristics and compaction quality monitoring [15,16,17]. These findings have provided solid guidance for optimizing design and construction procedures. However, during the operation stage, the sand foundation is susceptible to progressive loss due to long-term hydraulic erosion and cyclic loading, which can gradually compromise structural integrity [18]. This slow deterioration often results in differential settlement, stress concentration, and cracking, thereby diminishing the tunnel’s ability to withstand future disruptions—its resilience.

Resilience is a comprehensive index that characterizes a structure’s capacity to resist damage, sustain functionality, and recover from disturbances, making it a critical framework for evaluating infrastructure exposed to adverse conditions [19,20]. For immersed tunnels, resilience research has primarily focused on performance under extreme sudden events. Several studies have investigated seismic resilience through fragility analysis, vulnerability assessment, and restoration modeling [21,22,23]. Fire resilience has also attracted attention, particularly regarding user evacuation safety and post-accident traffic recovery strategies [24]. Additionally, the influence of water–soil coupling effects on stochastic seismic responses and reliability has been examined for immersed tunnels [25]. While these studies have advanced the understanding of immersed tunnel behavior under various hazards, they predominantly address short-term, high-intensity events rather than long-term progressive deterioration [26].

Beyond sudden hazards, the gradual evolution of tunnel resilience over extended service periods has gained increasing research interest, primarily in the context of shield tunnels. Huang and Zhang [27] established a resilience assessment framework for shield tunnel linings under extreme surcharge using horizontal convergence as the performance indicator. Lin et al. [28] extended this work by proposing an analytical model that accounts for multistage disturbances and recoveries, incorporating both convergence and settlement as performance metrics. Hua et al. [29] further developed a longitudinal resilience characterization method based on relative differential settlement, demonstrating the importance of axial deformation patterns. These studies have progressively refined resilience evaluation methods for shield tunnels under long-term service conditions. However, most of these resilience assessments rely on single deformation indicators, such as convergence or settlement, without adequately considering other critical damage metrics like cracking propagation, joint opening, and bearing capacity degradation [30].

Current monitoring for immersed tunnels mainly targets structural responses like deformation and internal forces, with limited capability to continuously assess sand foundation conditions [31]. Conventional methods, including manual inspection and periodic geophysical surveys, only provide discrete snapshots, failing to capture progressive sand loss and its gradual impact on structural performance. This impedes establishing the temporal link between foundation degradation and resilience deterioration, a topic rarely addressed in immersed tunnel research [32].

To address these gaps, this study focuses on an in-service immersed tunnel and develops a comprehensive framework to evaluate structural damage and resilience under sand foundation loss. This study begins with analyzing the condition of the sand foundation and the extent of structural damage using data from field monitoring and detection. To enable precise identification of sand foundation deterioration, a joint sensing system integrating the distributed optical fiber strain sensing (DOFS) network and impact imaging is established. Then, a refined three-dimensional numerical model that accounts for structural damage is established and validated against measured deformation data, followed by a comprehensive analysis of segment deformation, internal force redistribution, and crack propagation induced by sand foundation loss. Subsequently, a resilience grading framework based on redundancy quantification is proposed, which incorporates the damage evolution state and the existing structural bearing capacity redundancy, and the resilience grade of the tunnel is evaluated accordingly. This framework enables quantitative characterization of structural resilience evolution and provides a graded criterion for service performance assessment of immersed tunnels. Finally, the resilience evolution is predicted under various displacement recovery ratios, considering the damage caused by differential settlement accumulation, based on the established numerical model and resilience grading system. These findings provide a theoretical basis and technical support for structural damage detection and resilience-based repair decision-making for in-service immersed tunnels affected by sand foundation loss.

## 2. Project Overview

**Figure 1:**
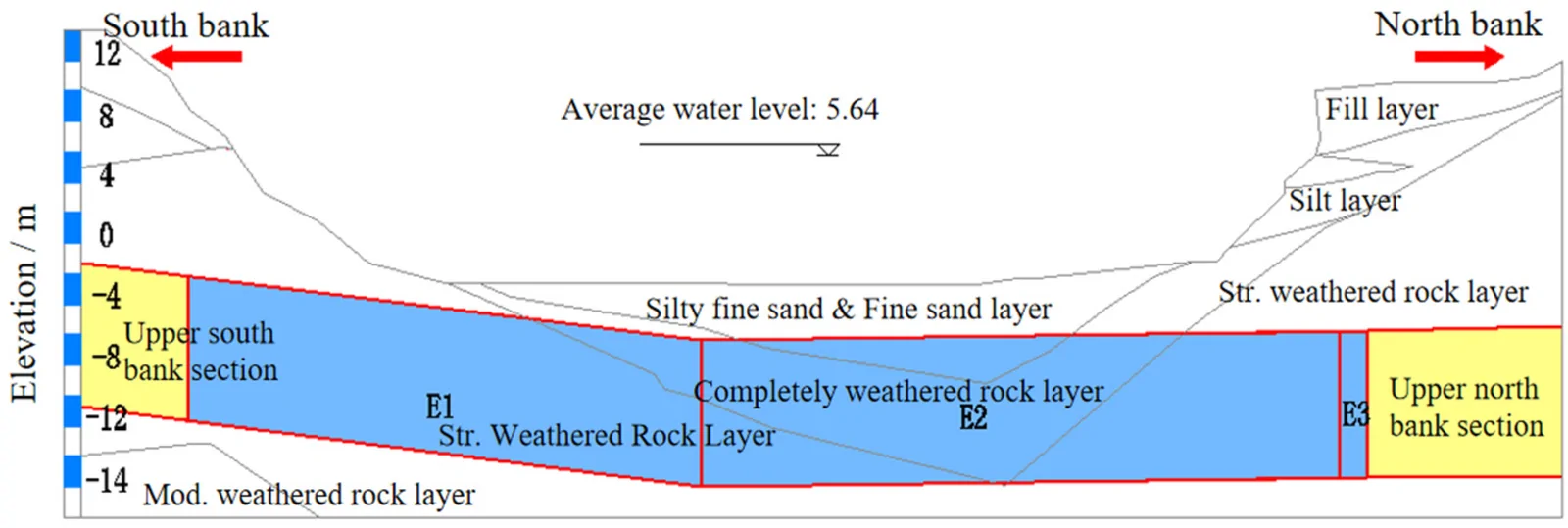
Longitudinal profile of the immersed tunnel (unit: m).

**Figure 2:**
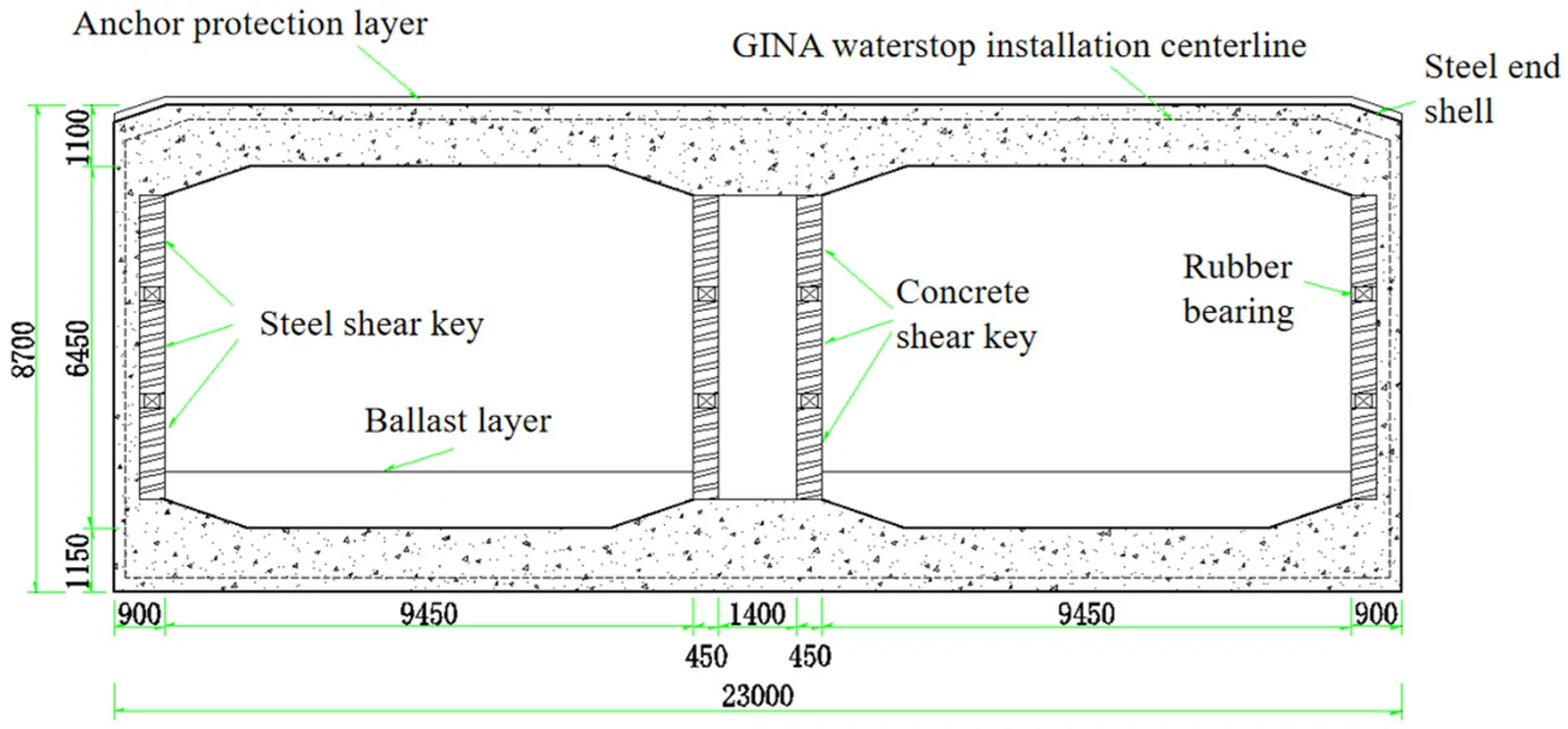
Typical cross-sectional view of the immersed tunnel (unit: mm).

The immersed tunnel studied in this paper has been in operation for over a decade. It is a two-way, four-lane tunnel, designed to meet the urban arterial road standard, with a design speed of 50 km/h. As shown in Figure 1, the immersed section of the tunnel comprises three segments: E1 (94.0 m), E2 (116.0 m), and E3 (4.0 m), running from the south bank to the north bank of the river. The total length of the immersed section is 214 m. This tunnel is a rectangular reinforced concrete structure, 23.00 m wide and 8.70 m high, as presented in Figure 2. The immersed section of the tunnel contains two traffic bores and a service gallery. The thicknesses of the base slab, roof slab, side walls, and central partition wall are 115 cm, 110 cm, 90 cm, and 45 cm, respectively. The joints between the segments are flexible and are equipped with vertical reinforced concrete shear keys (in the central partition wall), vertical steel shear keys (in the side walls), and horizontal reinforced concrete shear keys, which collectively resist shear forces and accommodate deformations. These immersed segments are founded on a 60 cm-thick sand foundation. Based on the geological survey data, the strata along the tunnel alignment are, from top to bottom, fill, silt, silty fine sand, completely weathered rock, strongly weathered rock, and moderately weathered rock.

## 3. In Situ Sensing System and Data Analysis

### 3.1. Structural Deformation Monitoring System

**Figure 3:**
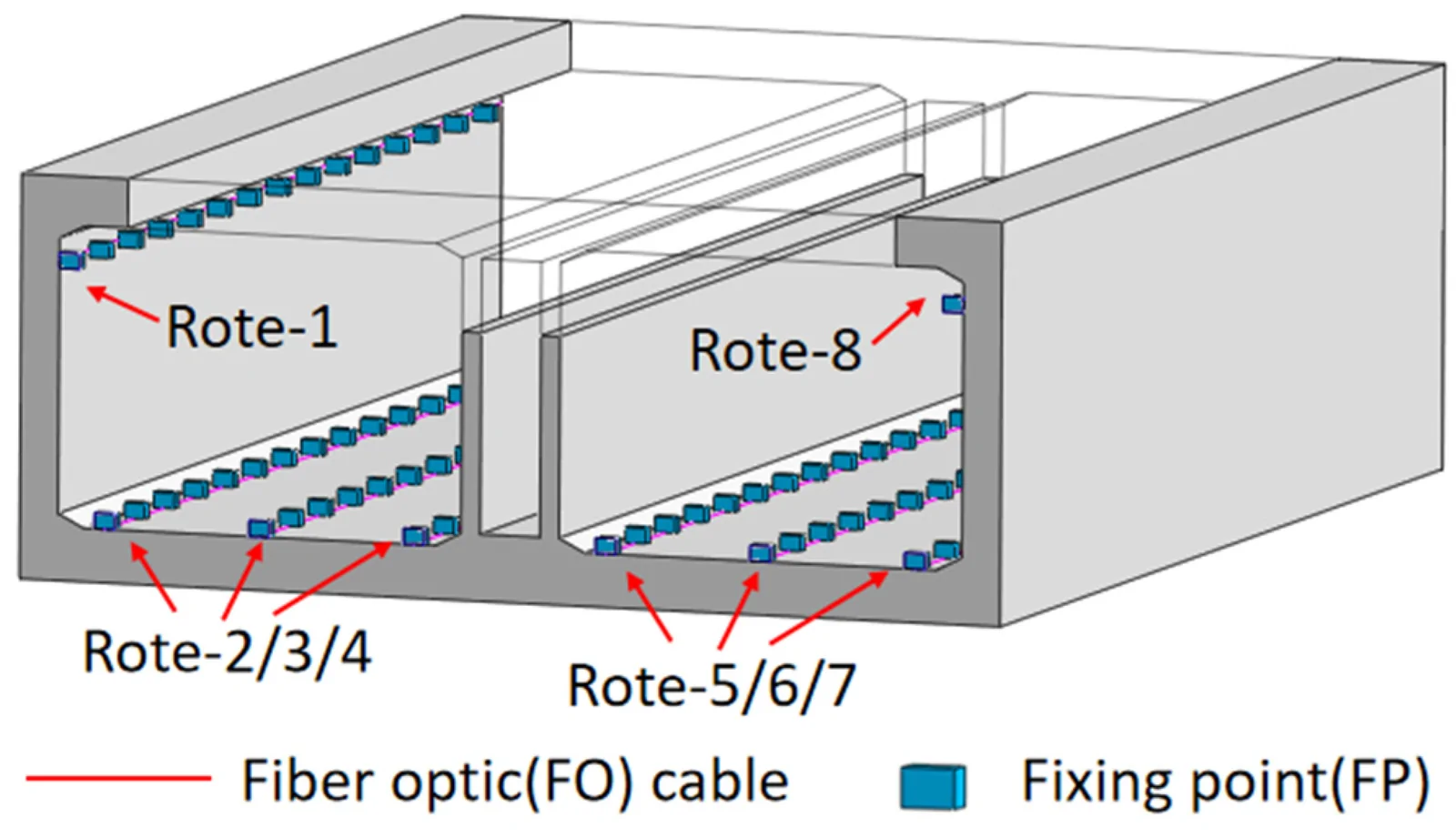
Layout of the DOFS-based deformation monitoring system.

To accurately obtain the long-term deformation characteristics of the tunnel structure, a distributed optical fiber strain sensing (DOFS) network was deployed along the tunnel during construction. The layout of this DOFS-based deformation monitoring system is illustrated in Figure 3. The sensing system consists of 8 long-gauge optical fiber cables, a high-precision optical fiber demodulator, and a data acquisition terminal. The optical fiber cables are embedded in the tunnel floor and side walls, covering all segment joints and key stress sections. The main technical parameters of the sensing system are as follows:

(1) Spatial resolution: 1 m;
(2) Sampling frequency: 1 Hz;
(3) Strain measurement accuracy: ±2 με;
(4) Settlement conversion accuracy: ±0.5 mm.

The system adopts temperature self-compensating optical fibers, which eliminate the influence of temperature changes in the tunnel on strain measurement through differential calculation between reference fibers and measurement fibers, with a temperature compensation accuracy of ±0.5 °C.

The DOFS system operates on the principle of Brillouin scattering, where the Brillouin frequency shift (BFS) Δν exhibits a linear relationship with both strain and temperature variation [33].

(1) $$\Delta v=C_{\varepsilon }\varepsilon +C_{T}\Delta T$$

where $C_{\varepsilon }$ and $C_{T}$ are the strain and temperature sensitivity coefficients, respectively. The system features temperature self-compensating fibers, enabling separation of strain and temperature effects. The strain-induced frequency shift is converted to strain via $\varepsilon =\Delta v/C_{\varepsilon }T$. With a 1 m spatial resolution, strain profiles are obtained along the tunnel at a 1 Hz sampling frequency. Vertical displacements $S\left(x\right)$ are derived through double integration of strain distribution [34].

(2) $$S\left(x\right)=\iint \varepsilon \left(x\right)dxdx+C_{1}x+C_{2}$$

where $C_{1}$ and $C_{2}$ are integration constants determined by boundary conditions.

### 3.2. Sand Foundation Defect Detection Method

The sand foundation filling condition was inspected using the impact imaging method based on elastic wave propagation in layered media. The detection system comprises a 5 kg impact hammer, 8-channel velocity geophones (natural frequency 100 Hz, channel spacing 0.5 m), and an IP68 waterproof acquisition terminal. Along the tunnel longitudinal direction, survey lines were arranged at 3.0 m intervals, covering the full 211 m segment with approximately 70 lines and 560 measuring points in total. The sampling interval was 0.125 ms (8 kHz) with a 0.1 s record length per test, providing a spatial resolution of 0.5 m and a detection depth of 0–5 m.

During data processing, raw signals were first denoised via wavelet thresholding, followed by parallel time-domain (response energy), frequency-domain (FFT, half-power bandwidth for frequency-dominant signals), and time-frequency-domain (CWT) analyses. The damping attenuation factor *R_f_*  is adopted as the core quantitative indicator, referring to the verified schemes from Chen [35]. For densely compacted media, the wave impedance difference between adjacent layers is relatively small, allowing elastic waves to propagate slowly with weak reflections and mild attenuation. In contrast, in loose or voided media, the significant wave impedance contrast causes elastic waves to travel faster, accompanied by strong waveform reflections and rapid energy attenuation. Therefore, by characterizing the attenuation behavior of elastic waves in conjunction with time-frequency domain signal analysis using the impact imaging method, the damping attenuation factor *R_f_* is proposed as a quantitative indicator for evaluating the compaction state of the sand foundation in immersed tunnels.

(3) $$R_{f}=\frac{f_{r}}{\Delta f}$$

where $f_{r}$ is the dominant elastic wave resonance frequency, and Δf is the half-power bandwidth of the frequency spectrum.

The quantitative relationship between *R_f_* and sand compaction state *D_r_* was established by Chen [35] through laboratory model tests and core-drilling validations, and the grading criteria for compaction states are summarized in Table 1. Reliability was verified by repeated tests showing a coefficient of variation < 5% for *R_f_*, and comparison with intrusive coring at 10% of the total points yielded a consistency rate of 92%. Regarding uncertainties, they primarily arise from underwater ambient noise, inconsistent hammer energy, and potential omission of small-scale voids due to the 0.5 m channel spacing; these were mitigated by multi-stack averaging, adaptive wavelet denoising, and combined time-frequency interpretation to distinguish defect reflections from boundary interferences.

### 3.3. Deformation of the Tunnel

**Figure 4:**
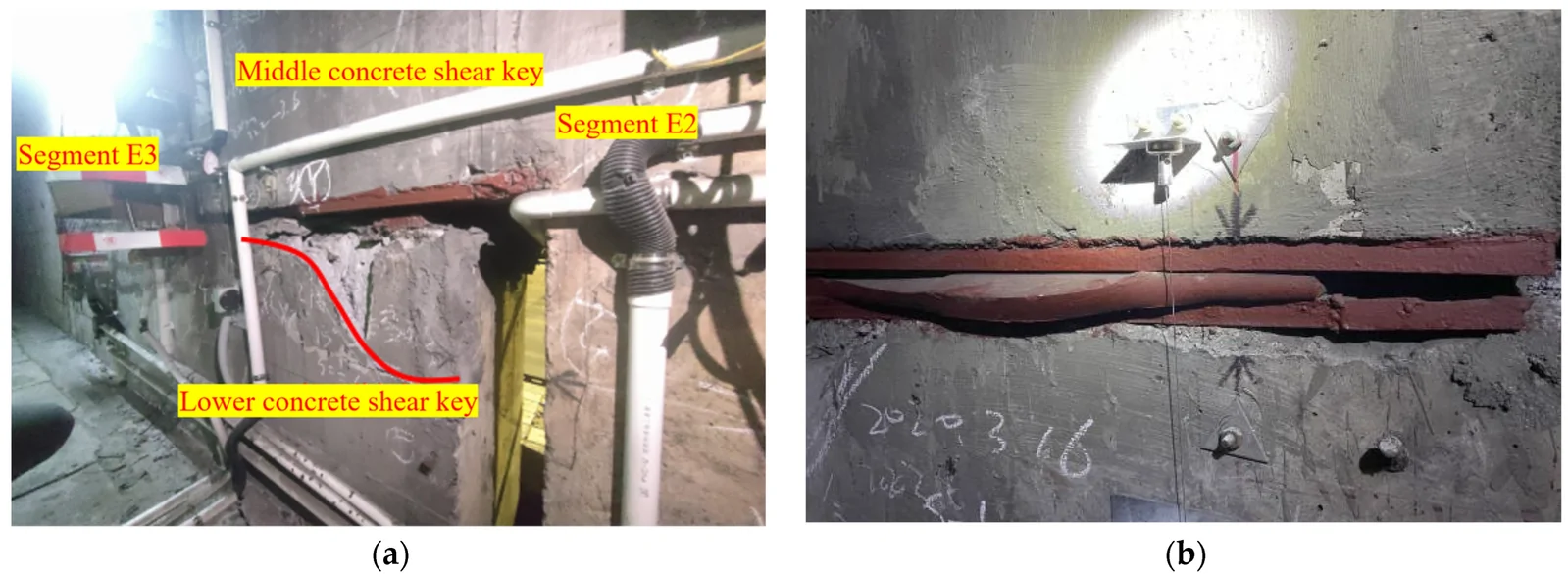
Defects at the E2–E3 segment joint: (**a**) Partial concrete spalling; (**b**) severe extrusion of the rubber bearing.

Field inspections were carried out to assess the condition and severity of damage to the tunnel structure while it was in service. As shown in Figure 4, defects at the tunnel joint were documented. Distinct cracking is evident in the pavement, crash barriers, and maintenance walkways within the E2–E3 joint region. In this region, the steel shear keys on the sidewalls are severely corroded, while the concrete shear keys on the central partition wall are significantly deformed and misaligned, indicating hard contact between adjacent shear keys. Furthermore, the rubber bearings associated with the shear keys are heavily compressed, with severe extrusion observed (Figure 4b).

**Figure 5:**
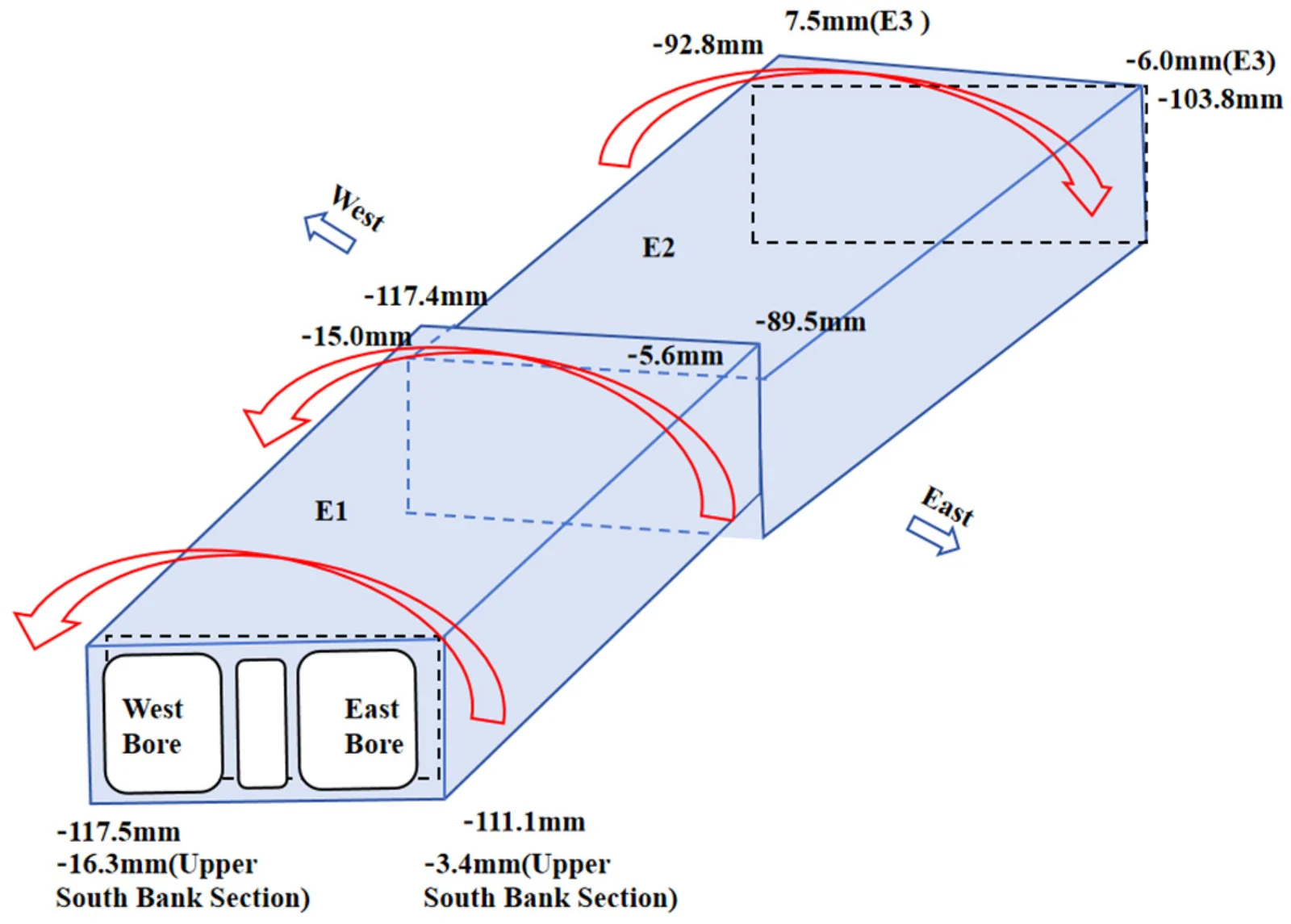
Schematic of the differential settlement of segment joints.

Based on the distributed optical fiber sensing and settlement monitoring data, the deformation modes of Segments E1 and E2 are plotted in Figure 5, which reveals the distinct deformation characteristics between these segments. As shown in Figure 5, Segment E2 exhibits significant flexural–torsional deformation, with its southern portion tilting westward and its northern portion tilting eastward. By contrast, Segment E1 exhibits consistent westward tilting across both the northern and southern sides, accompanied by considerably greater settlement on the southern side than on the northern side. Differential settlements measured on both the eastern and western sides of the southern end of Segment E1 are 107.7 mm and 101.2 mm, respectively. The differential settlement at the connection between the southern end of Segment E1 and the upper south bank section reaches 106.7 mm, which is the maximum differential settlement of the entire tunnel. For the northern end of Segment E2, the corresponding differential settlement values are 97.8 mm and 100.3 mm on the eastern and western sides. Thus, the differential settlement values on the eastern and western sides of the E1–E2 joint are 83.9 mm and 102.4 mm, respectively. The abnormal deformation areas reflected by the sensing data are consistent with the on-site inspection results of structural cracks and component extrusion.

According to the above field monitoring data and the Technical Specifications for the Management and Maintenance of Inland-River Immersed Tunnel [18], the service status of this tunnel’s structure is rated as Grade D. Consequently, traffic restrictions are required, and targeted major repairs or reinforcement must be carried out.

### 3.4. Compaction State of the Sand Foundation

**Figure 6:**
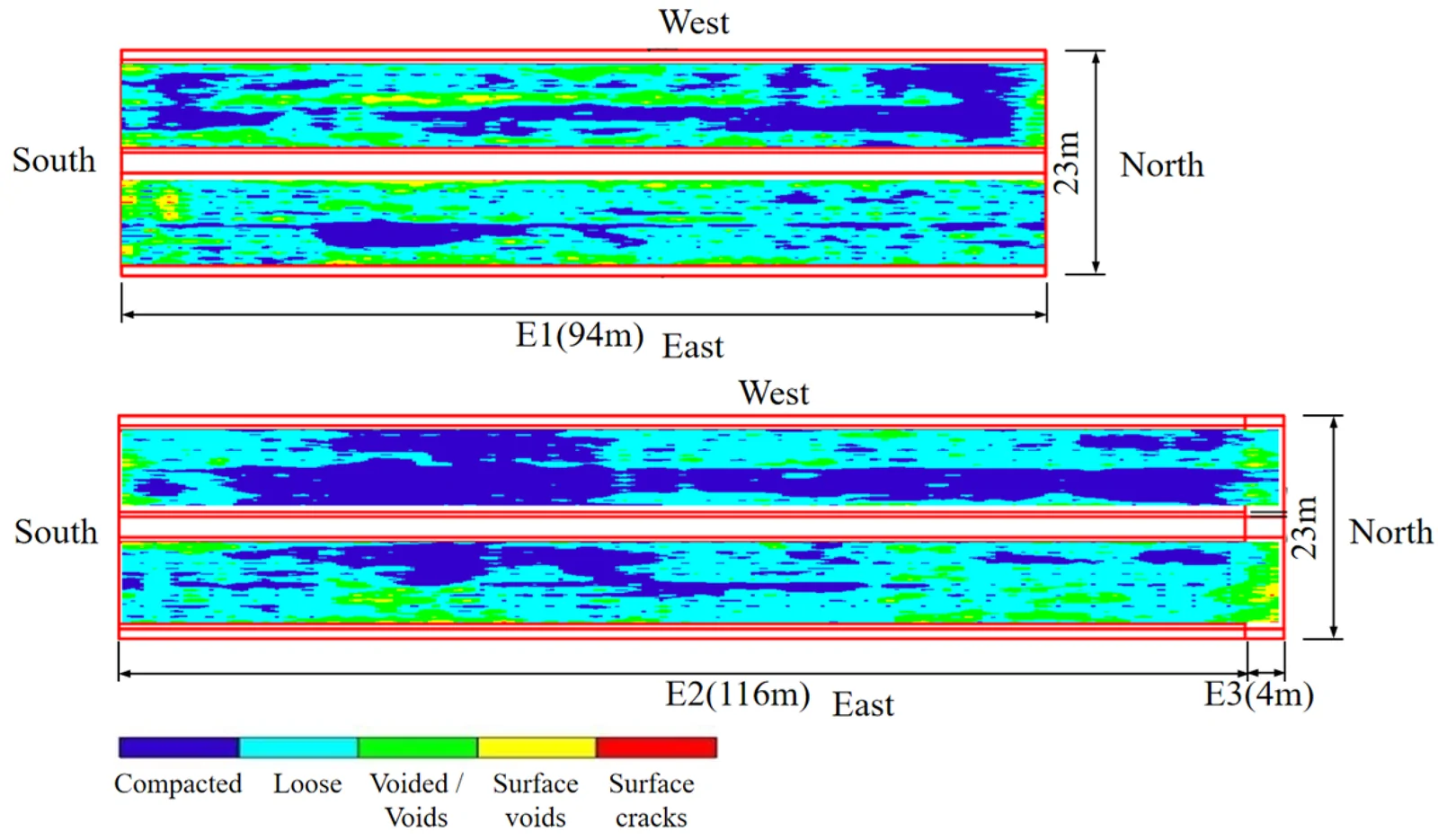
Sand foundation filling status for E1 and E2.

In field analysis, simple cross-verification with other elastic wave parameters is performed to reduce the interference of signal noise. The detection results are presented in Table 2 and Figure 6. A comprehensive analysis of the 8 characteristic parameters (3 time-domain response energies, dominant frequency, dominant frequency amplitude, secondary frequency, secondary frequency amplitude, and wavelet decay time) reveals that the total proportion of loose and voided areas exceeds 50%, indicating significant sand foundation loss. The detection results are mutually verified with the distributed optical fiber sensing data, which jointly reveal the spatial distribution characteristics and development trend of sand foundation loss.

As shown in Figure 6, the foundation beneath the western traffic bore of Segment E1 contains a large, relatively continuous compacted zone accompanied by several continuous voided zones. By contrast, the foundation of the eastern bore of Segment E1 is dominated by extensive loose sand, with a continuous voided zone along its southern side. The foundation of the western bore of Segment E2 comprises 50% continuously distributed compacted areas, alongside substantial continuous loose zones and localized voids. The eastern bore of Segment E2 mainly comprises a large, continuous loose region (45%), with local voiding.

The opposing tilting directions observed on the northern and southern sides of Segment E2 are attributed to the continuous void zones adjacent to the northern side of the eastern bore and the southern side of the western bore. Evidently, uneven loss of the sand foundation and the formation of continuous voids are the primary triggers of differential settlement, which has in turn inflicted severe damage to the segment structure, especially at the joints.

For the examined tunnel, damage assessment and investigation of resilience evolution following repair and resilience-enhancement measures are of considerable practical significance for developing effective repair strategies.

## 4. Mechanical Response of Tunnel Structure

### 4.1. Numerical Simulation Setup

**Figure 7:**
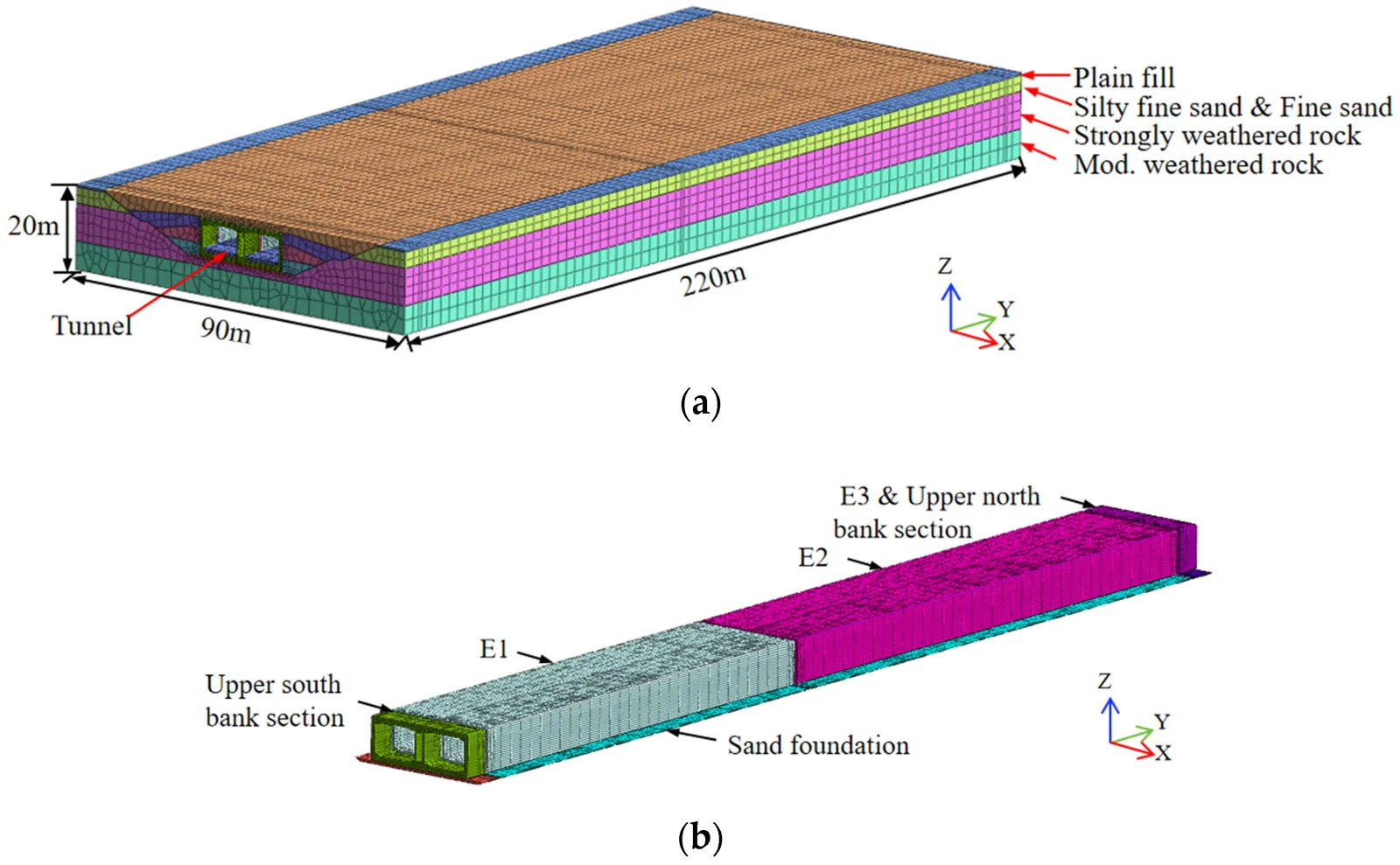
Three-dimensional solid finite element model: (**a**) Geometry and mesh of numerical model; (**b**) mesh of tunnel and its sand foundation.

A refined three-dimensional tunnel-stratum interaction model was established, as shown in Figure 7, and was used to analyze the tunnel response under sand foundation loss. To eliminate the boundary effect during numerical computation, the computational domain was sized at 220 m in the longitudinal (X) direction, 90 m in the transverse (Y) direction, and 20 m in the vertical (Z) direction. This was achieved while taking into account the site’s geological conditions and the immersed tunnel’s geometric dimensions. The model comprises approximately 30,000 three-dimensional solid elements (C3D8R) and 200,000 nodes. The sand foundation and the bottom surface of the tunnel share common nodes to ensure displacement continuity and force transmission at the interface. Boundary conditions are defined as fixed at the bottom, normal constraints on the lateral surfaces, and a free top surface. A mesh convergence analysis was conducted to ensure solution accuracy; the global element size is 0.5 m, with local refinements to 0.2 m at segment joints and shear keys. Boundary conditions were defined to minimize boundary effects: the bottom surface was fully fixed, while the four lateral surfaces were assigned roller constraints permitting in-plane movement. The top surface was left unconstrained vertically but subjected to hydrostatic pressure determined by the river water level above the tunnel.

During the numerical modeling process, solid elements were employed to represent both the soil and rock strata, the backfill layers, the tunnel segments, the shear keys, and the rubber bearings. Shell elements were applied to model the response of the steel end shells at segment joints and GINA waterstops in the immersed tunnel. Measurement plates were embedded within the segment structure to provide a convenient way to extract the internal forces of the tunnel segments when numerical analysis is conducted. The unit weight and elastic modulus of these measurement plate elements were set to 1/1000 of the actual structural values to minimize their influence on the global mechanical response of the model.

A concrete damaged plasticity (CDP) model was applied to the immersed tunnel segments. It can be used to characterize the impact of damage evolution on tunnel performance and quantify the evolution of resilience in the tunnel structure after repair and reinforcement. The parameters of the CDP model, determined by referring to the Code for Design of Concrete Structure [36] and references [26], are listed in Table 3, while the physical–mechanical parameters of the on-site soil and rock strata are provided in Table 4. During the numerical simulation, four simulation stages were conducted sequentially to accurately capture the response of the tunnel and sand foundation. These stages involve the application of initial geostatic stress, tunnel construction, initial operation, and sand foundation loss. The details of each stage are presented in Table 5. During simulations, the material properties of the sand foundation elements were degraded according to the gradation from “compacted” to “loose” to “voided” states reflecting the loss of bearing capacity and stiffness of the sand foundation.

**Figure 8:**
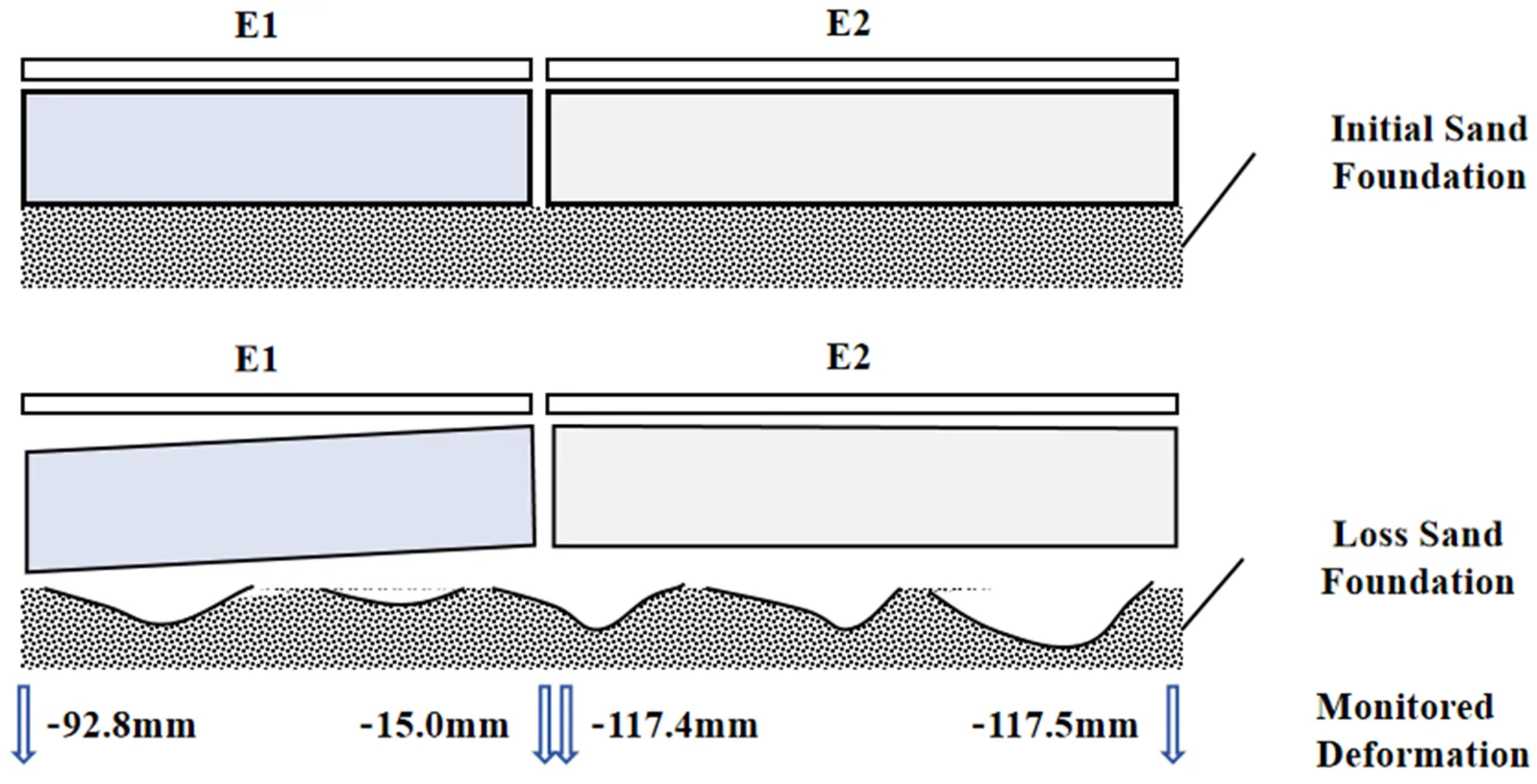
Schematic of sand foundation loss inducing differential displacement.

To simulate the differential settlement induced by sand foundation loss as shown in Figure 8, the state of the sand foundation was altered from its original compacted condition. Under the combined effect of the reduced foundation parameters and the aforementioned loads (a traffic load of 20 kPa and the siltation load), the resulting deformation pattern closely matched the monitoring data. In the initial operation stage, the sand foundation was maintained in the “compacted” state with these loads applied. For the sand foundation loss stage, the same traffic and siltation loads were retained, while the foundation parameters were assigned the “void” state values from Table 4 to replicate the loss scenario depicted in Figure 8.

### 4.2. Model Validation

**Figure 9:**
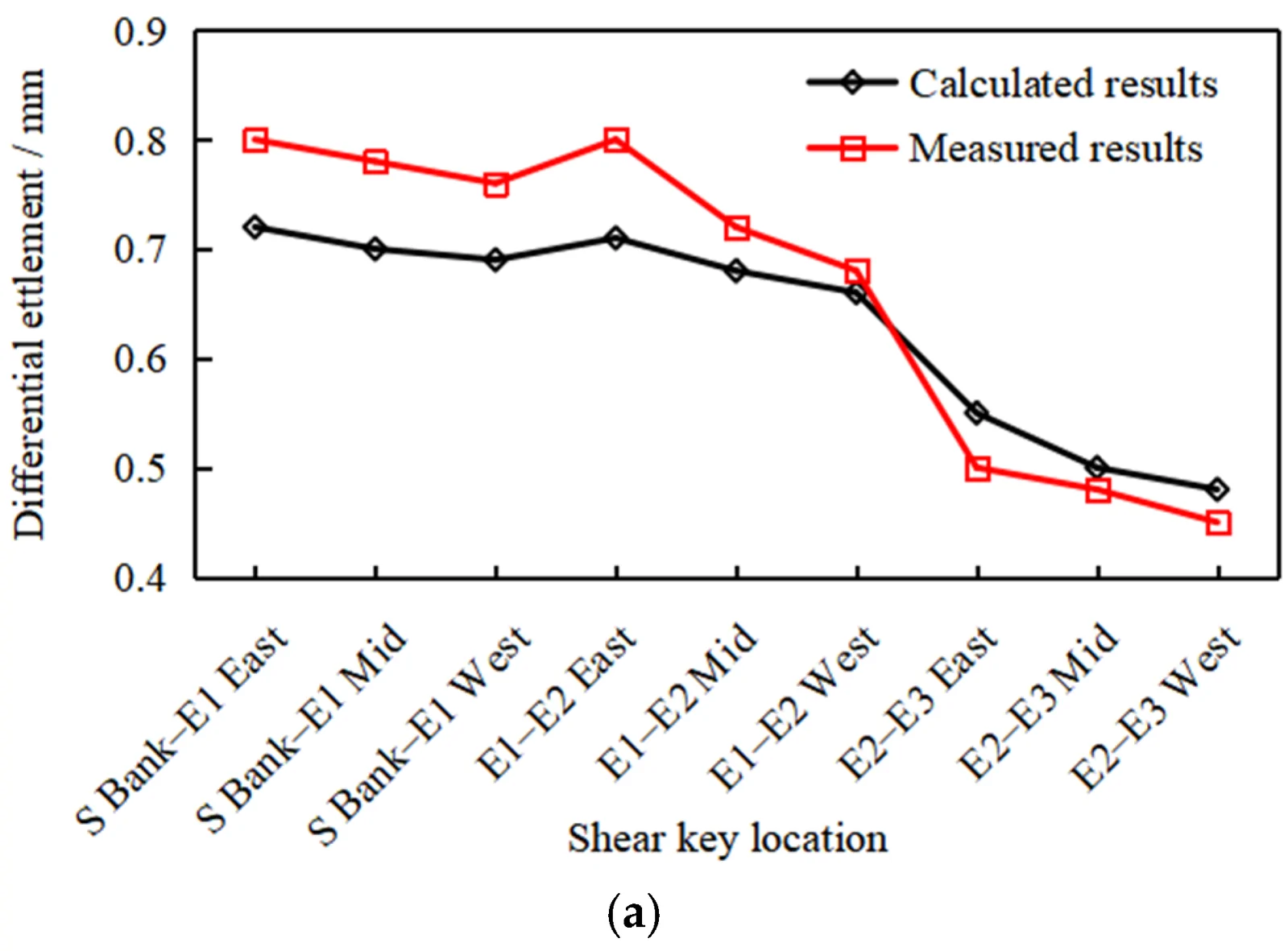

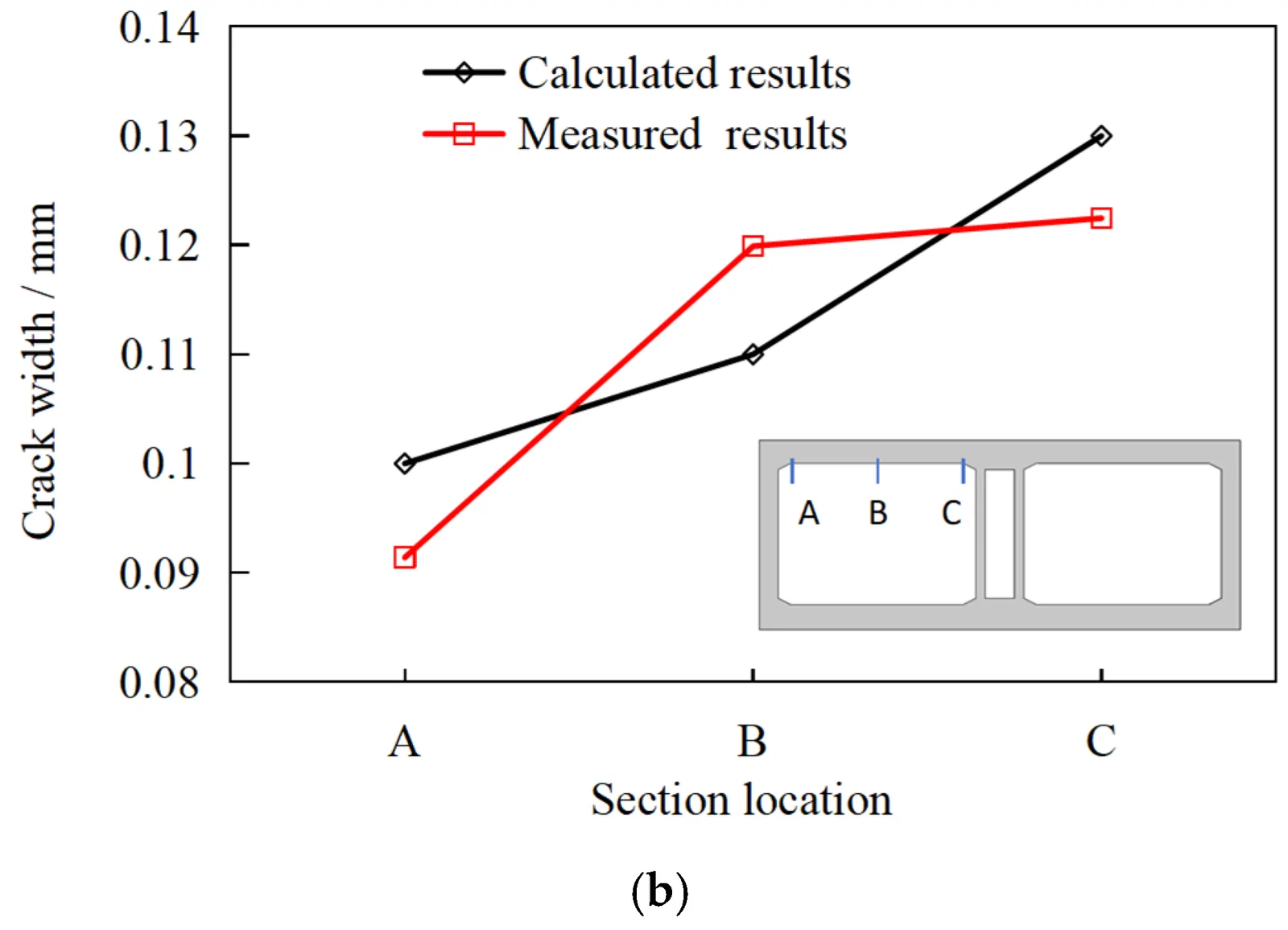
Validation of the numerical model: (**a**) comparison between simulation deformation and monitoring data, (**b**) comparison between calculated crack width and field-measured data.

To validate the above numerical model, the differential settlements obtained from the numerical analysis of the initial operation phase were compared with distributed optical fiber sensing data, as shown in Figure 9a. The results show that the computed differential settlements at the South Bank-E1, E1–E2, and E2–E3 joints are consistent with the measured data. The maximum relative error is only 9%, confirming that the numerical simulation accurately reproduces the tunnel’s actual mechanical behavior.

To further validate the numerical model, the field-measured crack widths of the studied tunnel structure at the initial operation stage were also employed as an additional response quantity for comparison. Using the bending moments obtained from the numerical model, crack widths were calculated following the method prescribed in the Code for Design of Concrete Structures [36] (detailed in Section 4.5). Figure 9b presents a comparison between the calculated and measured crack widths at the top of the side wall, middle wall, and mid-span (points A, B, and C in Figure 9b). The maximum discrepancy is 9.4%, confirming that the numerical model can effectively capture the actual mechanical deformation response of the tunnel structure.

### 4.3. Analysis on Displacement Distribution

**Figure 10:**
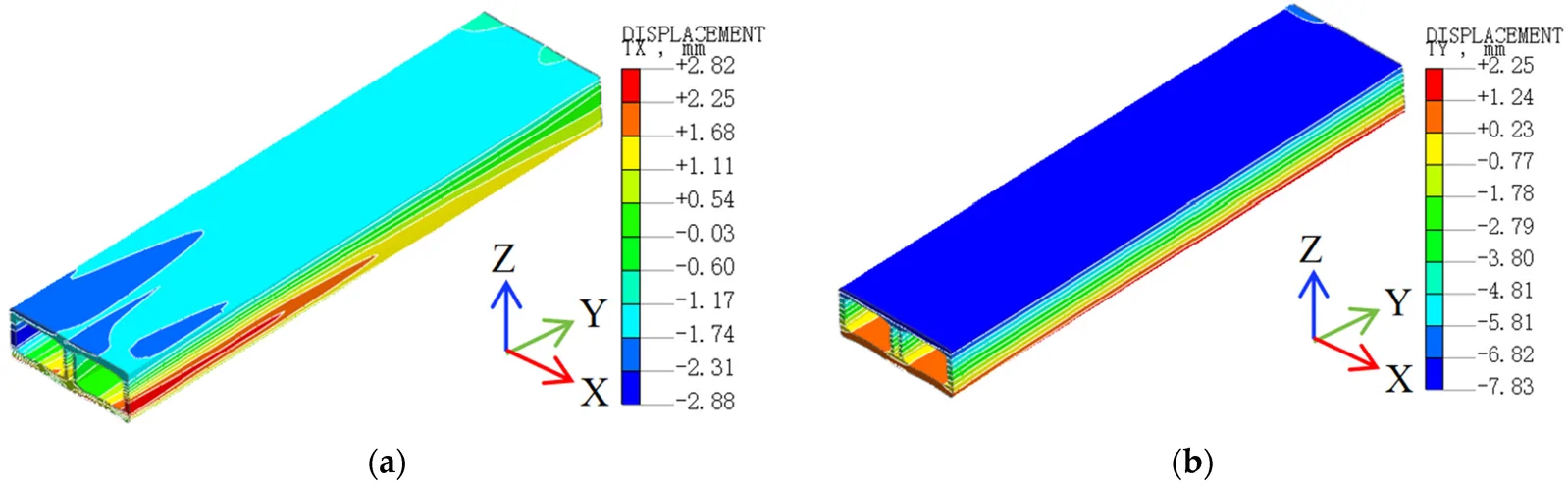

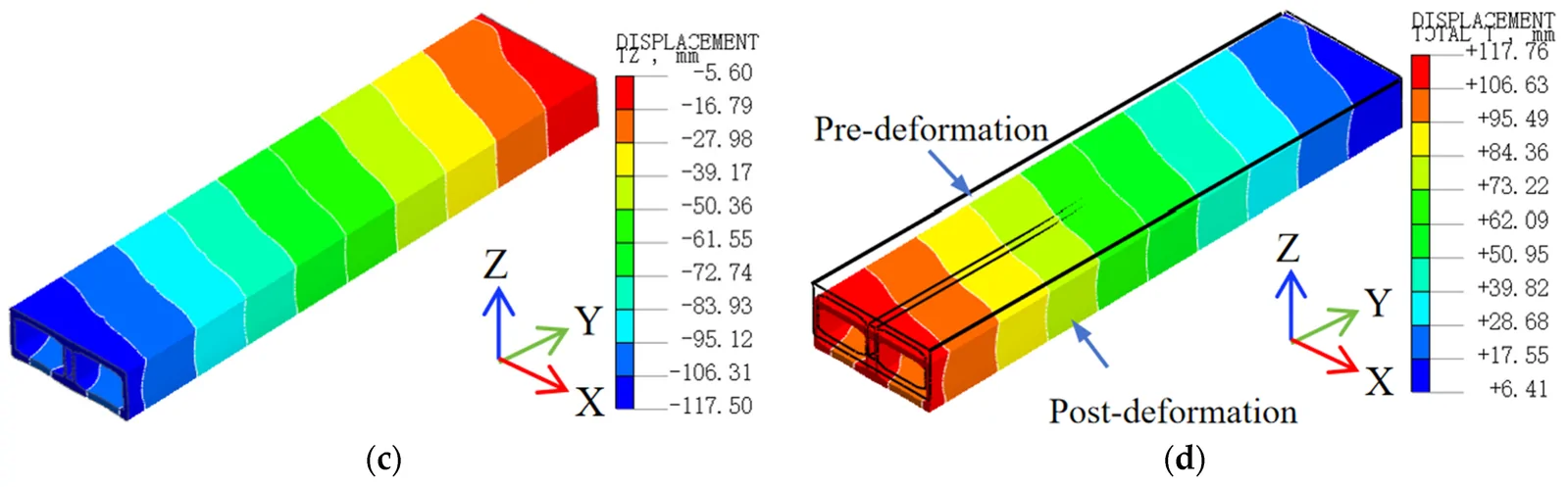
Distribution of the displacement of Segment E1: (**a**) X-direction; (**b**) Y-direction; (**c**) Z-direction; (**d**) Total displacement.

**Figure 11:**
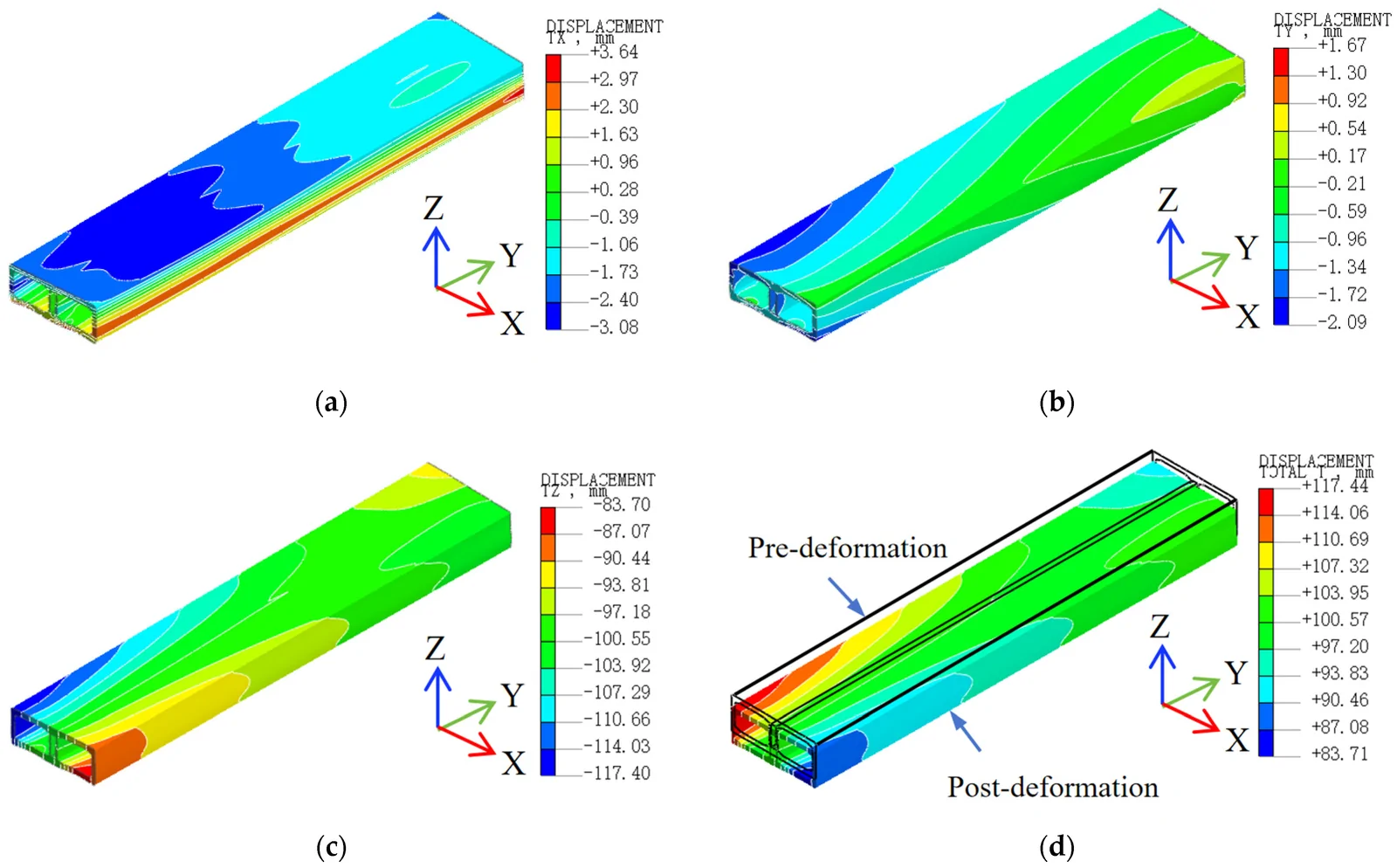
Distribution of the displacement of Segment E2: (**a**) X-direction; (**b**) Y-direction; (**c**) Z-direction; (**d**) Total displacement.

The displacement distributions in the X-, Y-, and Z-directions of Segments E1 and E2 obtained by the validated numerical model are shown in Figure 9 and Figure 10, respectively. Due to the coupling effects of external loads (e.g., traffic, siltation, and hydrostatic pressure) and the loss of the sand foundation, significant settlement and horizontal displacements occur in both segments. As presented in Figure 10, the maximum horizontal displacement and settlement of Segment E1 are 7.83 mm and 117.50 mm, respectively, whereas the maximum horizontal displacement and settlement of Segment E2 are 3.64 mm and 117.40 mm, as shown in Figure 11. The deformation distribution across both segments is highly non-uniform. Specifically, Segment E1 tilts as a whole towards the southwest. In contrast, Segment E2 sinks overall and exhibits opposite tilting between its southern (westward) and northern (eastern) sides, resulting in distinct bending–torsional deformation. Thus, the differential settlement and bending-torsional deformation caused by the loss of the sand foundation directly induce internal force redistribution and severe stress concentration in the tunnel structure.

### 4.4. Analysis on Bending Moment Distributions

**Figure 12:**
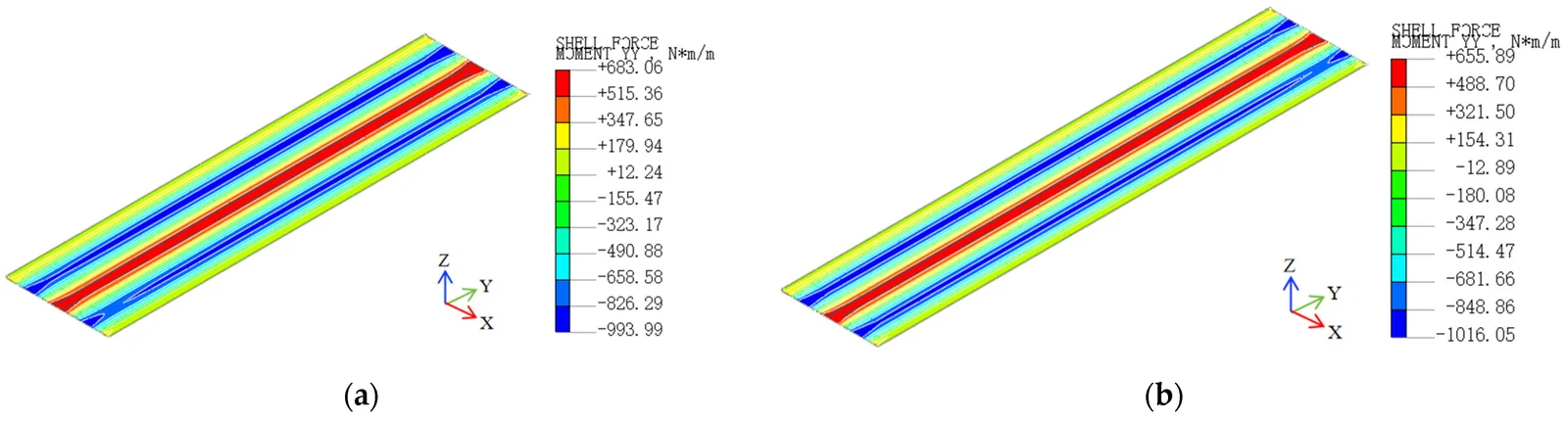
Bending moment distribution in the bottom slab during the initial operation stage (×1000): (**a**) Segment E1; (**b**) Segment E2.

**Figure 13:**
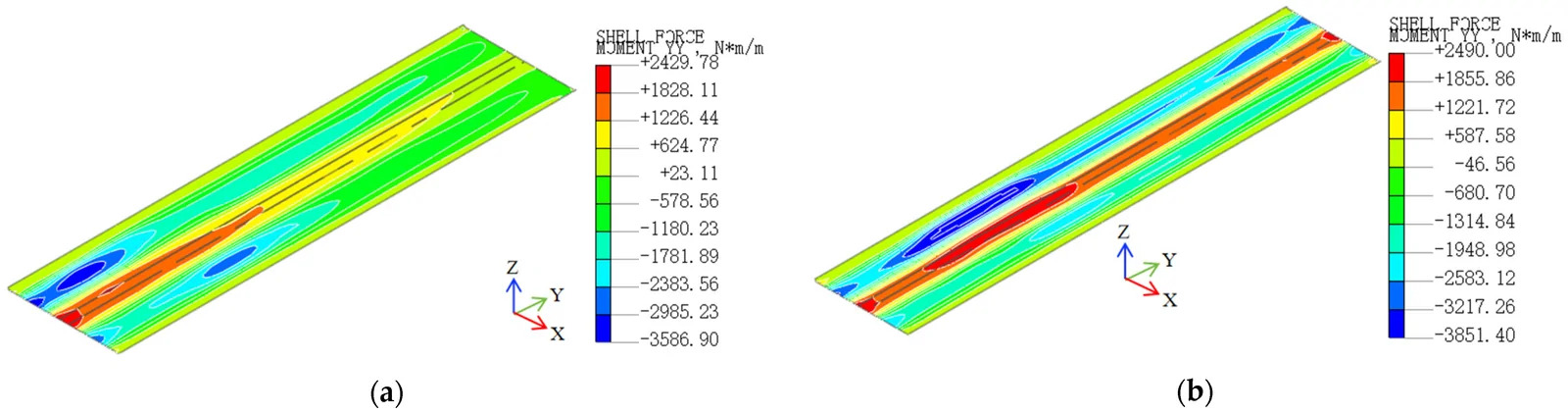
Bending moment distribution in the bottom slab during the sand foundation loss stage (×1000): (**a**) Segment E1; (**b**) Segment E2.

The bending-moment distributions in the bottom slabs of Segments E1 and E2 during the initial operation and sand foundation loss stages are shown in Figure 12 and Figure 13, respectively. In this section, the bending moment is defined as positive when the inner side of the slab is under tensile stress and as negative when the outer side is under tensile stress.

As shown in Figure 12, the distribution of bending moments remains uniform in both base slabs during the initial operating stage. Under the coupling effects of self-weight and siltation loads, the eastern and western bores of the base slabs in Segments E1 and E2 are subjected to tension, while the outer side of the central service gallery is also under tension. The maximum positive bending moment is 683.06 kN·m, occurring at the mid-span of the central section of Segment E1. Meanwhile, the maximum negative bending moment is 1016.05 kN·m, which occurs at the mid-span of the eastern side of Segment E2.

As shown in Figure 13, the distribution of bending moments in the base slabs becomes highly non-uniform during the sand foundation loss stage. This clearly indicates the significant redistribution of internal forces induced by foundation degradation. The mechanical responses of the two slabs differ markedly. Specifically, Segment E1 undergoes severe bending–torsional deformation due to excessive settlement at the southern end, which leads to a significant increase in the bending moment in local areas. Meanwhile, Segment E2 experiences overall subsidence due to extensive voiding in the eastern sand foundation. The western compacted zone bears most of the structural loads, resulting in severe local bending moment concentration. At this stage, the maximum positive bending moment is 2490.00 kN·m at the mid-span of the central gallery of Segment E2, which is 2.64 times greater than in the initial operation stage, when the sand foundation was intact and compacted. Similarly, the maximum negative bending moment is 3851.40 kN·m at the mid-span of the western bore of Segment E2, which is 2.79 times greater than in the initial operation stage when the sand foundation was intact and compacted. These findings suggest that regions with a residual compacted sand foundation are highly susceptible to stress concentration, which aggravates uneven internal force distribution within the tunnel structure following the loss of sand foundation.

### 4.5. Structural Damage Assessment

To quantify the damage state of the immersed tunnel, the maximum crack width is used to evaluate the damage evolution of tunnel segments. In accordance with the Code for Design of Concrete Structures [36], the maximum crack width of the tunnel structure can be calculated using Equations (4)–(7):

(4) $$\omega_{\max}=\alpha_{cr}\psi \frac{\sigma_{s}}{E_{s}}\left(1.9c_{s}+0.08\frac{d_{eq}}{\rho_{te}}\right)$$

(5) $$\psi =1.1-0.65\frac{f_{tk}}{\rho_{te}\sigma_{s}}$$

(6) $$d_{eq}=\frac{\sum n_{i}d_{i}^{2}}{\sum n_{i}v_{i}d_{i}},$$

(7) $$\rho_{\mathrm{te}}=\frac{A_{s}+A_{p}}{A_{te}}$$

where *α_cr_* is the characteristic coefficient for the member under force and is taken as 1.9 for flexurally reinforced concrete members; *ψ* is the non-uniformity coefficient of steel strain; *σ_s_* is the stress in the tensile reinforcement; *E_s_* is the elastic modulus of the reinforcement and is taken as 200 GPa; *c_s_* is the distance from the outer edge of the outermost tensile bar to the surface of the concrete and is taken as 40 mm; *ρ_te_* is the reinforcement ratio used in the calculation; *A_te_* is the effective area of concrete in tension; *A_s_* is the cross-sectional area of longitudinal ordinary reinforcement in the tension zone; *A_p_* is the cross-sectional area of longitudinal prestressed reinforcement in the tension zone; *d_eq_* is the equivalent diameter of the longitudinal reinforcement in the tension zone; *d_i_*, *v_i_*, and *n_i_* are the nominal diameter, relative bond characteristic coefficient, and number of the i-th type of longitudinal reinforcement in the tension zone, respectively.

Sections exhibiting the maximum bending moment during the initial operation stage (Stage 3) and the sand foundation loss stage (Stage 4) were selected for crack width calculation using Equations (4)–(7). As shown in Figure 9b, during the initial operation stage, the calculated maximum crack width was 0.13 mm (measured: 0.122 mm), which fell below the design control limit of 0.2 mm. This calculated value was consistent with field monitoring data. In contrast, during the sand foundation loss stage, the calculated maximum crack width increased significantly to 0.43 mm based on the simulated internal forces, far exceeding the standard control limit of 0.2 mm [36]. Although this 0.43 mm is a model-derived value rather than a direct field measurement, the simulated crack width aligns well with field observations of severe distress, including concrete spalling, shear key misalignment, and rubber bearing extrusion (Figure 4). This consistency confirms the model’s capability to capture the actual damage state, despite the calculated crack width surpassing the code-specified limit.

A comprehensive analysis of deformation, bending moment and crack width shows that the tunnel is currently in a critically defective state. Its deformation resistance and load-bearing capacity have deteriorated significantly, necessitating urgent repairs and reinforcement.

## 5. Resilience Grading Framework and Evolution Prediction

### 5.1. The Redundancy Factor and Resilience Grading Framework

To date, resilience assessment methods specifically tailored to immersed tunnels under sand foundation loss remain lacking. Relevant concepts and methodologies can be referred to and adapted from those developed for other types of tunnels (e.g., shield tunnels [28,29]); however, the distinct structural characteristics of immersed tunnels, including the segmental joints and the flexible sand foundation, necessitate context-specific modifications. Since bearing capacity serves as the foundation for achieving other resilience dimensions under sand foundation loss conditions, this study starts from this fundamental dimension to establish a tailored resilience evaluation framework.

Therefore, a redundancy factor (*T*) is proposed in this study, integrating multiple indicators including crack width, damage state, and bearing capacity redundancy, to provide a more comprehensive evaluation of resilience evolution for immersed tunnels subjected to progressive sand foundation loss.

(8) $$T=\frac{F_{u}-F_{d}}{F_{u}-F_{r}}$$

where *F*_u_ is the ultimate bearing capacity of the intact structure; *F*_r_ is the ultimate bearing capacity of the damaged structure; and *F*_d_ is the design bearing capacity of the intact structure. For reinforced concrete flexural members, damage and cracking may result in a reduction in the effective depth of the cross-section [37]. This reduced effective depth can then be substituted into the formula for calculating the flexural capacity of the damaged structure to determine its ultimate bearing capacity (*F***_r_**).

Foundation loss in immersed tunnels is a progressive deterioration process that gradually induces loss of foundation support, redistribution of internal forces, crack propagation, joint damage, and ultimately bearing capacity degradation. Unlike sudden extreme events, this slow cumulative mechanism does not produce a clear onset of failure or a well-defined recovery trajectory, and conventional resilience frameworks that rely on assumed performance recovery curves become less applicable. The redundancy factor *T* in Equation (8) directly quantifies the remaining bearing capacity margin, providing a physically grounded metric that captures the essence of this deterioration mechanism.

The grading thresholds for *T* are established based on: (i) the crack width limits prescribed by the Code for Design of Concrete Structures (GB 50010-2010) [36], (ii) resilience grading concepts from existing literature [27,38,39], and (iii) long-term monitoring and inspection data from multiple operational immersed tunnels, including the subject tunnel investigated in this paper. The strong alignment between these code provisions, field measurements, and the proposed grading criteria ensures that the framework is not purely theoretical but is firmly rooted in engineering practice. For instance, under the sand foundation loss condition analyzed in Section 4, the subject tunnel exhibits a redundancy factor of *T* = 1.59 with a calculated crack width of 0.43 mm, corresponding to a low resilience grade. As the redundancy factor decreases with increasing crack width and progressive damage, the grading framework consistently captures the physical deterioration process observed in both numerical results and field inspection data, thereby demonstrating its rationality and practical applicability for evaluating resilience evolution in immersed tunnels subjected to sand foundation loss. Table 6 summarizes the proposed resilience grading criteria, providing a quantitative basis for resilience assessment and repair decision-making under sand foundation loss conditions.

### 5.2. The Displacement Recovery Ratio

The foregoing analysis confirms that the case-study tunnel has insufficient structural resilience, severely undermining its safe operation. Accordingly, targeted retrofitting measures are required to mitigate differential settlement caused by the deterioration of the sand foundation and enhance the tunnel’s overall structural resilience, thereby guaranteeing its long-term operational safety. To analyze the deterioration of the sand foundation and the grouting process, as well as changes in structural resilience throughout the repair process, the existing 3D numerical model was adopted to simulate resilience evolution under various displacement recovery ratios (*R*). Displacement recovery ratio (*R*) is defined as follows:

(9) $$R=\frac{S_{\mathrm{mr}}-S_{\mathrm{cr}}}{S_{\mathrm{mr}}}\times 100\%$$

where *S_mr_* is the maximum residual displacement of the tunnel, and *S_cr_* is the current residual displacement of the tunnel.

It should be noted that the displacement recovery ratio *R* serves as a comprehensive indicator to characterize the effectiveness of repair interventions. In engineering practice, grouting is commonly adopted to fill the voids caused by sand foundation loss, and the combined effect of grouting pressure and subsequent slurry hardening can further facilitate the uplift of deformed tunnel segments and the correction of differential settlement. However, the diffusion mechanism of grout in underwater sand foundation is extremely complex, influenced by multiple factors including grouting pressure, slurry properties, soil permeability, and the spatial distribution of pre-existing voids. Establishing a reliable quantitative relationship between grouting parameters and the resulting recovery ratio under such conditions remains a challenging task that is beyond the scope of this study. Therefore, this paper focuses on the resilience evolution and grading of the immersed tunnel following various recovery levels, rather than investigating the influencing factors of the recovery ratio itself. The practicality of these recovery scenarios can be further validated through field monitoring and adaptive adjustment during actual implementation.

Based on the previously derived calculation method for the redundancy factor (*T*), the following procedures are used to predict the resilience evolution after repair:

(1) Simulate the mechanical response of the tunnel structure under different displacement recovery ratios based on the calculated results at the sand foundation loss stage, taking into account the impact of structural damage on resilience.
(2) Calculate the crack width of the structure in the relevant states using Equations (4)–(7), and calculate the crack depth according to the method in Reference [37].
(3) Solve for the ultimate bearing capacity of the damaged structure, Fr, under each state.
(4) Substitute the results into Equation (8) to calculate the redundancy factor, establish the resilience evolution curve, and conduct resilience grading according to the standard in Table 6.

### 5.3. Evolution of Maximum Bending Moment Versus R

**Figure 14:**
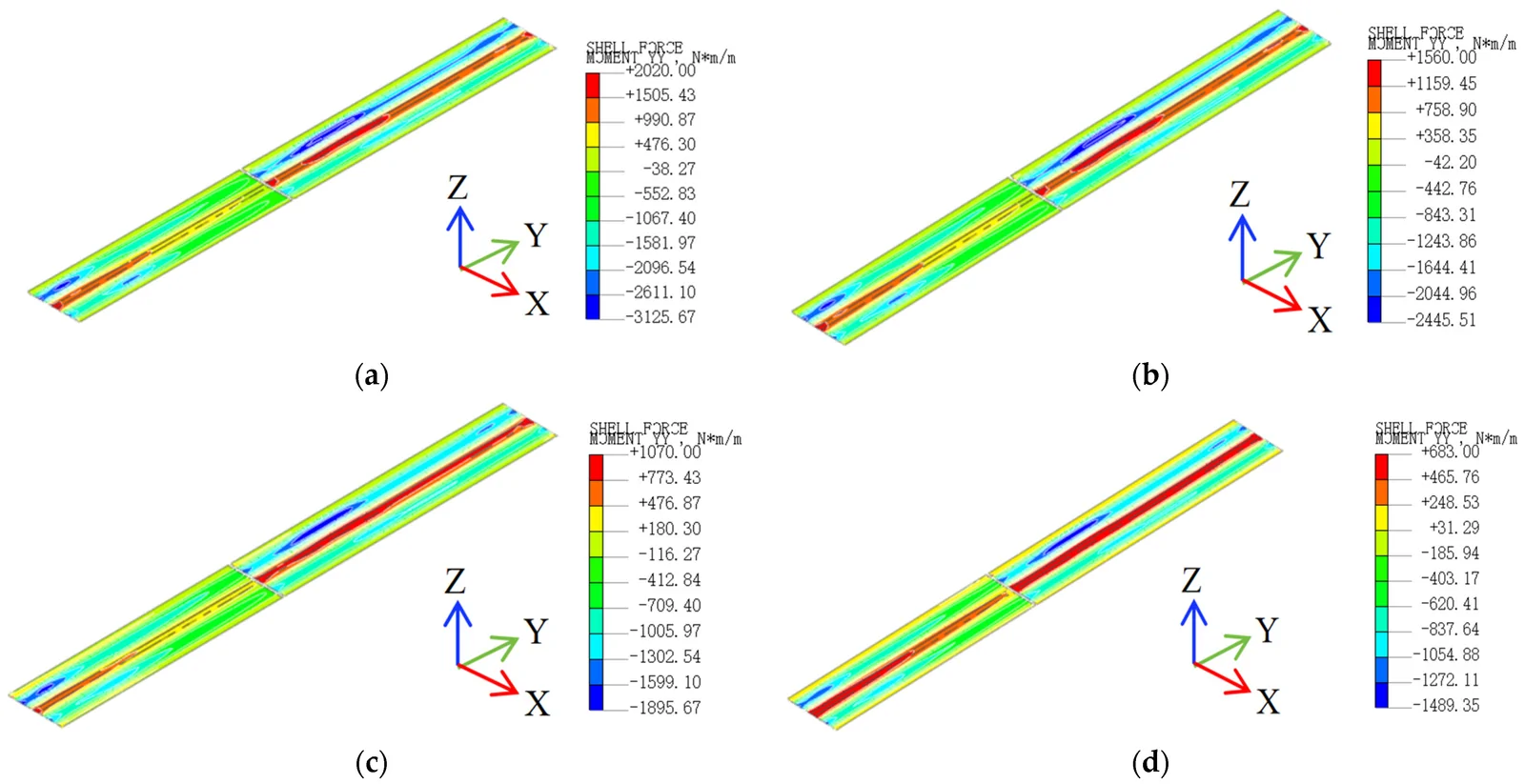
Bending moment distribution of bottom slabs under different displacement recovery ratios: (**a**) recovered by 20%; (**b**) recovered by 40%; (**c**) recovered by 60%; (**d**) recovered by 80%.

Based on the above numerical model, simulations were conducted to obtain the bending moment distributions on the bottom slabs for various displacement recovery ratios, ranging from 20% to 80%, as illustrated in Figure 14. Similar distribution patterns emerge across the different recovery ratios, with the maximum negative bending moment consistently occurring at the mid-span of the western bore of Segment E2. Overall, peak bending moments decrease steadily as the recovery ratio increases, indicating that displacement recovery effectively alleviates the internal force concentration.

**Figure 15:**
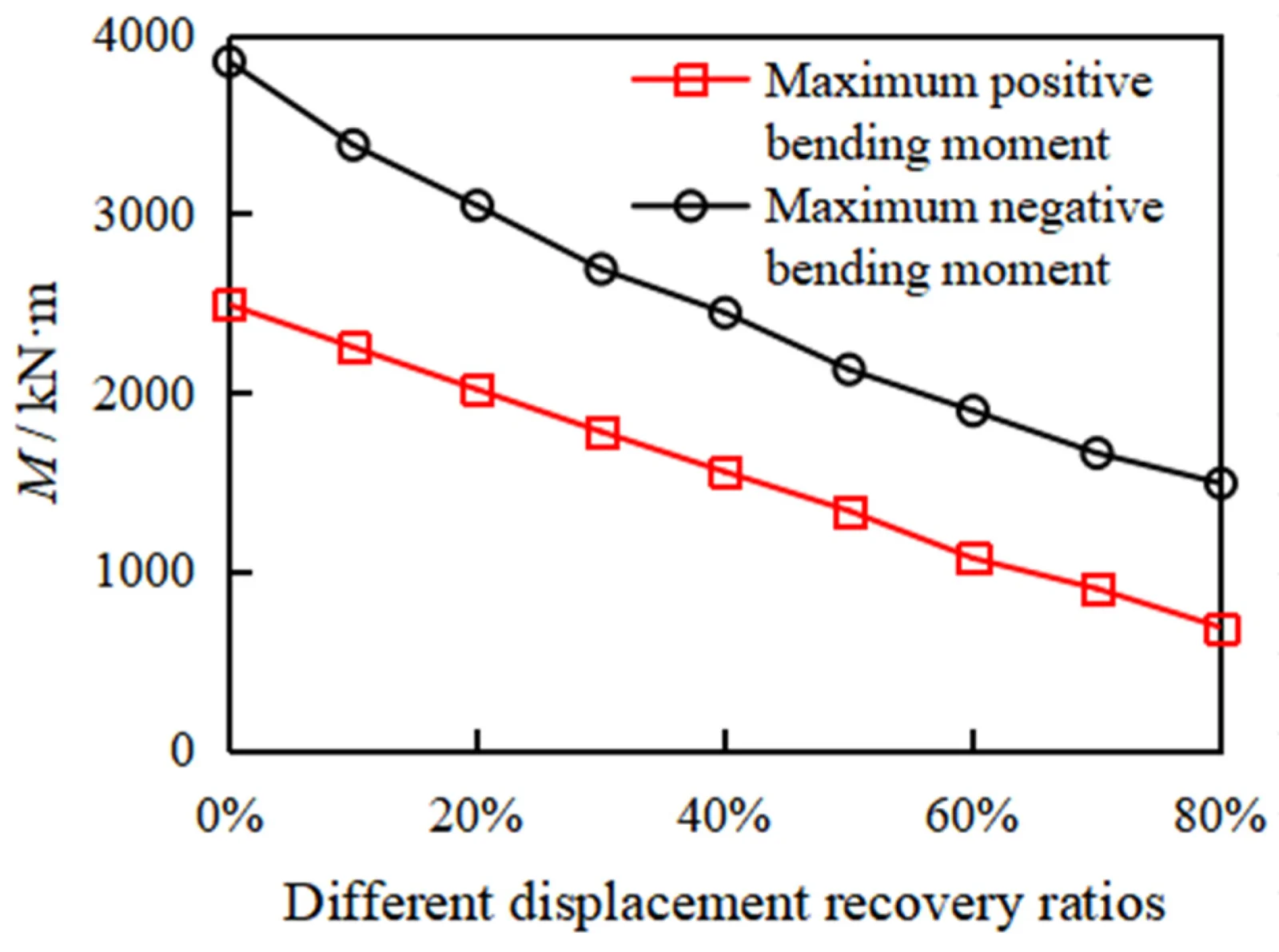
Maximum positive/negative bending moments vs. displacement recovery ratio.

Based on numerical simulations under displacement recovery, Figure 15 presents the variation in the maximum bending moment with the displacement recovery ratio. Specifically, the maximum positive bending moment decreases approximately linearly. This trend closely correlates with the mechanical performance of the positive bending zone in the central mid-span gallery of Segment E2. With a relatively modest slab span, this section exhibits low bending moment levels under identical foundation reactions and remains purely elastic with negligible plastic damage. Consequently, internal force redistribution remains stable and linear with respect to displacement recovery. In contrast, the mid-span region of Segment E2 has undergone plastic deformation due to negative bending. Accumulated plastic strain disrupts the linear transmission of internal forces, resulting in a nonlinear decrease and nonlinear unloading characteristics for the maximum negative bending moment.

### 5.4. Evolution of Crack Width and Redundancy Factor Versus R

**Figure 16:**
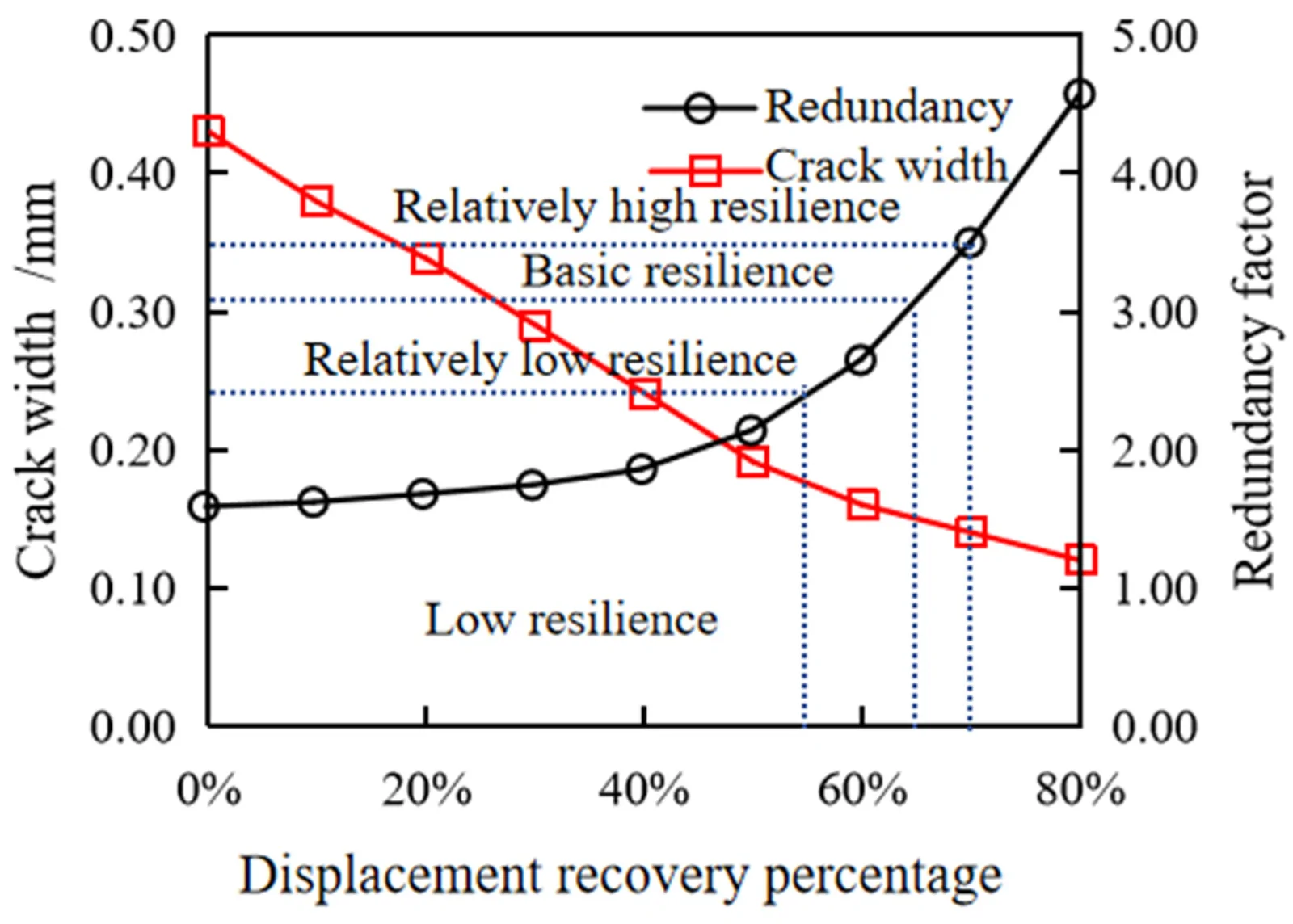
Evolution of both the redundancy factor and crack width.

The evolution of crack width and the redundancy factor under different displacement recovery ratios was investigated using the crack width calculation method in the literature [35] and Equation (7). The variations in the redundancy factor and crack width with the displacement recovery ratio are shown in Figure 16. As the displacement recovery ratio increases, the crack width decreases approximately linearly, while the redundancy factor *T* first exhibits a linear growth trend and then transitions into a parabolic growth pattern. This trend reflects the structural stress redistribution and resilience enhancement resulting from the progressive remediation of sand foundation defects and the simultaneous recovery of differential settlements. The mechanical response of the tunnel bottom slab is governed by two primary factors: (1) the formation of a “free surface” beneath the bottom slab due to sand foundation loss, which creates a free span (defined as the effective length of the bottom slab section losing direct support from the underlying sand layer) that induces substantial bending moments under self-weight and operational loads; and (2) additional internal stresses induced by differential settlements between adjacent segments. The coupling effects of the free span and additional stress caused by differential deformation control the overall structural behavior. When the displacement recovery ratio is below 50%, the free span remains relatively large, and the additional stresses from differential settlements are still pronounced due to limited deformation recovery. Consequently, the redundancy factor *T* increases only moderately. Once the recovery ratio exceeds 50%, two concurrent mechanisms take effect: on the one hand, differential settlements are largely recovered, significantly reducing the associated additional stresses; on the other hand, the progressive infilling of the sand foundation gradually shortens the free span. As the free span decreases to less than the bottom slab thickness, the bending moments diminish markedly. These coupling effects lead to a rapid growth of *T*, transitioning from a linear to a parabolic trend.

At a recovery ratio of 70%, the maximum crack width derived from the peak bending moment decreases to 0.14 mm, and *T* reaches 3.62, meeting the high resilience requirement defined in Table 6. It should be noted that the specific 70% threshold is obtained from numerical analyses for the case-study tunnel and is associated with its particular concrete strength grade, sectional dimensions, and reinforcement layout, and is therefore directly applicable to immersed tunnels with similar foundation types, structural forms, and hydro-geological conditions. Nevertheless, the five-tier resilience grading system established in Table 6—including the threshold values of the redundancy factor, allowable crack width limits, and corresponding resilience grades—is developed based on the provisions of design codes [36] and measured data from in-service immersed tunnels, and is thus supported by clear mechanical principles and engineering data. Consequently, this grading framework can be applied to the resilience assessment of various immersed tunnels. For different projects, the redundancy factor can be calculated based on the specific design bearing capacity and sectional properties, and the resilience grade can then be determined by referring to the criteria in Table 6.

## 6. Conclusions

This study uses an in-service immersed tunnel project as its research subject and systematically investigates structural damage assessment and resilience evolution prediction under sand foundation loss. This is achieved by integrating distributed optical fiber sensing data, impact imaging detection data, three-dimensional numerical simulations, and resilience evaluation methods. The main conclusions are outlined as follows:

(1) The DOFS-based field monitoring and impact imaging reveal that over 50% of the sand foundation area is affected by loosening or voids, which induce a bending-torsional deformation mode in the tunnel segments with a maximum joint differential settlement of 106.7 mm.
(2) Sand foundation loss causes significant internal force redistribution, increasing the maximum positive and negative bending moments to 2.64 and 2.79 times their initial values, with a calculated maximum crack width of 0.43 mm and a redundancy factor of 1.59, indicating a low resilience grade of the studied tunnel.
(3) The redundancy factor exhibits a parabolic growth pattern with a turning point at 50% displacement recovery, below which the increase is modest due to free span-induced bending moments and differential settlement-induced stresses, and above which it accelerates as the free span shortens and differential settlements are largely recovered, leading to a marked reduction in internal forces.
(4) At 70% displacement recovery, the crack width decreases to 0.14 mm and the redundancy factor reaches 3.62, achieving the high resilience grade; the proposed redundancy factor and grading framework provide quantitative criteria for resilience improvement strategies.

## Figures and Tables

**Table 1:** Quantitative grading criteria for sand foundation compaction state based on resistance factor *R_f_* [35].

*R_f_* Range	Compaction State	Engineering Implication
*R_f_* ≤ 3.0	Compacted	Meets design compaction requirements with stable bearing capacity
3.0 < *R_f_* ≤ 9.0	Loose	Insufficient compaction, prone to long-term uneven settlement
*Rf* > 9.0	Voided	Internal voids present, leading to poor bearing capacity and structural risks

**Table 2:** Statistical results of sand foundation filling status.

Location	Condition	E1	E3 + E2
East Side	Compacted	37%	34%
	Loose	39%	45%
	Voided	24%	21%
West Side	Compacted	48%	50%
	Loose	38%	32%
	Voided	14%	18%

**Table 3:** Parameters of the concrete damaged plasticity model.

Parameters	Values	Parameters	Values
Mass density *ρ* (kg/m^3^)	2500	Flow potential eccentricity *€*	0.1
Elastic modulus *e* (GPa)	34.5	*f*_bo_/*f*_co_	1.16
Poisson’s ratio *υ*	0.2	*F*_t,r_ (MPa)	2.64
Invariant stress ratio *k*_c_	0.667	*ε*_t,r_ (με)	110.08
Dilation angle (°)	30	*f*_c,r_ (MPa)	32.4
Viscosity parameter *μ*	0.0005	*ε*_c,r_ (με)	1678.4

**Table 4:** Parameters for soil layers and structural components.

Material Type	Mass Density (kN/m^3^)	Cohesion (kPa)	Friction Angle (°)	Elastic Modulus (MPa)	Poisson’s Ratio
Plain fill	17.6	10	10	12	0.3
Silty fine sand/fine sand	18.5	2.4	18	9.5	0.35
Completely weathered rock	20	26	20	15.5	0.3
Strongly weathered rock	21	28	26	70	0.28
Moderately weathered rock	24	33	36	280	0.22
Rubber pad	15	/	/	900	0.38
General backfill	16.5	12	11	15	0.2
Rubble backfill	17.2	5	20	18	0.3
Crushed stone filter layer	18.5	8	25	25	0.25
Sand cushion (compacted)	16.4	5	35	30	0.32
Sand cushion (loose)	15.4	3	20	20	0.32
Sand cushion (voided)	14.8	2	12	15	0.32

**Table 5:** Details on simulation conditions.

No.	Conditions	Brief Description
1	Initial geostatic stress analysis	Initial in situ stress balanced, displacement reset
2	Tunnel construction	Tunnel excavation and construction, displacement reset
3	Initial operation	Apply traffic load (20 kPa) and siltation load
4	Sand foundation loss	Modify the properties of the sand foundation to simulate sand foundation loss

**Table 6:** Resilience grading standard for immersed tunnels.

Fortification Objective	Resilience Grade	Damage Level	Safety and Durability Function		Redundancy Factor *T*	Description
			Structural Stability	Crack Width		
I	High resilience	Intact	Crack width meets normal design requirements, and the structure is entirely elastic	≤0.1 mm	≥5	Excellent resistance and load-bearing capacity
II	Relatively high resilience	Slight damage	Crack width meets normal design requirements, no progressive collapse	0.1~0.2 mm	3.5~5	Good resistance and load-bearing capacity
III	Basic resilience	Light damage	Obvious local cracking, individual sections enter plastic state, no progressive collapse	0.2~0.3 mm	3~3.5	Fair resistance and load-bearing capacity
IV	Relatively low resilience	Moderate damage	A few sections enter plastic state with severe cracking, no progressive collapse	0.3~0.4 mm	2.5~3	Poor resistance and load-bearing capacity
V	Low resilience	Severe damage	Structure enters plastic state with severe cracking, no progressive collapse	>0.4 mm	<2.5	Very poor resistance and load-bearing capacity

Notes: While the numerical sequence of the redundancy factors aligns with prior studies [27,38,39], the thresholds and corresponding damage descriptions in this table have been physically re-defined for immersed tunnels. The crack widths listed are model-derived values based on the validated numerical simulations presented in Section 4.

## Data Availability

The original contributions presented in the study are included in the article, further inquiries can be directed to the corresponding author.

## References

[1] Lunniss R., Baber J. (2013). Immersed Tunnels.

[2] Su C., Che A., Xu Z., Han Z., Han Z., Zhu F., Hai T. (2024). Mechanical behavior of immersed tunnel under the influence of differential settlements: A case study of GZ tunnel. Tunn. Undergr. Space Technol..

[3] Xie X., Yi C., Li W., Fang Y. (2019). Safety analysis of settlement monitoring data of joints of Yongjiang immersed tube tunnel during operation period. Chin. J. Geotech. Eng..

[4] Li J., Ni P. (2025). Assessment of joint behaviour of immersed tunnel with prefabricated push-out closure joint under differential settlement. Can. Geotech. J..

[5] Hu Z., Sun Y., Jiang L., Mao H., Xu Y., Luo Y. (2025). Waterproof performance and failure mechanism of gina gasket subject to diverse joint deformations: A case study of the yuliangzhou immersed tunnel. Case Stud. Constr. Mater..

[6] Chou Y. (2016). Deterioration assessment of an immersed-tube road tunnel in taiwan. Proc. Inst. Civ. Eng.-Forensic Eng..

[7] Xu X., Tong L., Liu S., Li H. (2019). Evaluation model for immersed tunnel health state: A case study of honggu tunnel, Jiangxi province, China. Tunn. Undergr. Space Technol..

[8] Li W., Fang Y., Mo H., Gu R., Chan J., Wang Y., Feng D. (2014). Model test of immersed tube tunnel foundation treated by sand-flow method. Tunn. Undergr. Space Technol..

[9] Xu Z., Che A., Su C. (2024). Evaluation on the state of sand filling layer and the influence on segment deformation of immersed tunnels. Undergr. Space.

[10] Grantz W. (2001). Immersed tunnel settlements. Part 1: Nature of settlements. Tunn. Undergr. Space Technol..

[11] Liu P., Chen J., Chen Y., Wang J. (2020). Mechanical model for joints of immersed tunnel considering the influence of joint differential settlement. Int. J. Geosynth. Ground Eng..

[12] Hu Z., Xie Y. (2016). Mechanical and failure characteristics of shear keys on immersed tunnel segment joints under differential settlements. Procedia Eng..

[13] Li G., Klingbeil L., Zimmermann F., Huang S., Kuhlmann H. (2020). An Integrated Positioning and Attitude Determination System for Immersed Tunnel Elements: A Simulation Study. Sensors.

[14] Chen J., Jiang X., Yan Y., Lang Q., Wang H., Ai Q. (2022). Dynamic Warning Method for Structural Health Monitoring Data Based on ARIMA: Case Study of Hong Kong-Zhuhai-Macao Bridge Immersed Tunnel. Sensors.

[15] Wang S., Zhang X., Bai Y. (2020). Comparative study on foundation treatment methods of immersed tunnels in China. Front. Struct. Civ. Eng..

[16] Che A., Zhu R., Fan Y., Feng S. (2019). An impact imaging method for monitoring on construction of immersed tube tunnel foundation treated by sand-filling method. Tunn. Undergr. Space Technol..

[17] He H., Lin Y., Li J., Zhang N. (2018). Immersed tunnel foundation on marine clay improved by sand compaction piles. Mar. Georesour. Geotechnol..

[18] (2019). Technical Specifications for the Management and Maintenance of Inland-River Immersed Tunnel.

[19] Tong J., Zhang W., Li C., Yuan D., Lou H., Wang Q., Wang H., Zhai J. (2024). Review of resilience evaluation methods in operational highway tunnel: Analysis of index systems and evaluation frameworks. J. Adv. Transp..

[20] Huang H., Zhang D., Huang Z. (2022). Resilience of city underground infrastructure under multi-hazards impact: From structural level to network level. Resilient Cities Struct..

[21] Huang Z., Zeng N., Zhang D., Argyroudis S., Mitoulis S. (2025). Resilience Models for Tunnel Recovery after Earthquakes. Engineering.

[22] Jiang J., Xun Z., Bai X., Liu D., Zhao K., Du X. (2024). Seismic damage mechanics and vulnerability analysis for the immersed tunnel subjected transverse earthquake records. Soil Dyn. Earthq. Eng..

[23] Jiang J., Chen X., Wang W., Zhao K., Du X. (2026). Seismic vulnerability analysis of submarine immersed tube tunnel under the mainshock-aftershocks. Soil Dyn. Earthq. Eng..

[24] Caliendo C., Genovese G., Russo I. (2022). A simultaneous analysis of the user safety and resilience of a twin-tube road tunnel. Appl. Sci..

[25] Wang Z., Luo Q., An J., Wang B. (2025). Stochastic seismic responses and seismic reliability assessment of the immersed tunnel considering the coupling effect of water-soil. Comput. Geotech..

[26] Zheng Y., Wu K., Wang L., Jiang Y., Liu Y. (2024). Structural damage assessment and failure mode analysis for cross-fault submarine tunnels. Eng. Fail. Anal..

[27] Huang H., Zhang D. (2016). Resilience analysis of shield tunnel lining under extreme surcharge: Characterization and field application. Tunn. Undergr. Space Technol..

[28] Lin X.-T., Chen X., Su D., Han K., Zhu M. (2022). An analytical model to evaluate the resilience of shield tunnel linings considering multistage disturbances and recoveries. Tunn. Undergr. Space Technol..

[29] Hua Y., Huang H., Zhang D. (2024). Longitudinal structural resilience of shield tunnel: Characterization and field application. Transp. Geotech..

[30] Han K., Zhang D., Chen X., Su D., Ju J., Lin X., Cui H. (2023). A resilience assessment framework for existing underground structures under adjacent construction disturbance. Tunn. Undergr. Space Technol..

[31] He S., Tang C., Pan M., Zhou W. (2025). Settlement-based triple factor framework for long-term safety assessment of immersed tunnel. Tunn. Undergr. Space Technol..

[32] Zheng G., Cheng X., Zhou H., Diao Y., Zeng C. (2022). Resilient evaluation and control of geotechnical and underground engineering structures. China Civ. Eng. J..

[33] Zhang X., Broere W. (2023). Monitoring seasonal deformation behavior of an immersed tunnel with distributed optical fiber sensors. Measurement.

[34] Wijaya H., Rajeev P., Gad E. (2021). Distributed optical fibre sensor for infrastructure monitoring: Field applications. Opt. Fiber Technol..

[35] Chen R. (2016). Nondestructive Testing for the Density of an Immersed Tunnel Foundation by the Sand-Flow Method. Mod. Tunn. Technol..

[36] (2015). Code for Design of Concrete Structures.

[37] Zhang J., Li Y., Wang W. (2018). Experimental study on correlation between crack width and depth of reinforced concrete beams. J. Disaster Prev. Mitig. Eng..

[38] Zheng G., Cheng X., Zhang Y. (2014). Simulation of progressive collapse and redundancy of ring-beam supporting structures of excavations. Chin. J. Geotech. Eng..

[39] Cheng X., Pei H., Yi F., Zheng G., Zhang Y., Ma Y., Wang S. (2024). Study on the mechanism and countermeasures of progressive collapse in deep excavation retained by multilayer struts. Structures.

